# Engineering Electron Transfer Flux between Cytochrome P450 Enzyme and P450 Reductase to Enhance Serotonin Production in *Escherichia Coli*

**DOI:** 10.1002/advs.202414859

**Published:** 2025-05-23

**Authors:** Wenzhao Xu, Pengling Wei, Lirong Chen, Ling Gao, Xiaole Xia

**Affiliations:** ^1^ Key Laboratory of Industrial Biotechnology Ministry of Education School of Biotechnology Jiangnan University 1800 Lihu Road Wuxi Jiangsu 214122 China

**Keywords:** cytochrome P450 enzymes, electron transfer flux, P450 reductases, RNA‐biosensors, serotonin, tryptamine‐5‐hydroxylases, whole‐cell biocatalysts

## Abstract

Microbial cell factories produce valuable compounds by exploiting cytochrome P450 catalytic systems. However, the inefficient electron transfer flux (ETF) between P450 and cytochrome P450 reductase (CPR) hinders the efficient synthesis of natural products. Herein, an ETF is systematically engineered by regulating the electron transfer rate, electron‐receiving rate, and electron donor NADPH availability for serotonin production. First, a putative electron transfer pathway (ETP) is identified using virtual computing and evolved based on a genetically encoded serotonin RNA biosensor. Subsequently, an intermediate site strategy is developed to shorten the electron‐hopping steps and distance in the ETP of CPR for enhancing the electron transfer rate. Next, the heme‐binding domain is engineered to reduce the distance between heme‐Fe and the substrate channel terminal in T5H for improving the electron‐receiving rate. Furthermore, the NADPH pool is enlarged to increase the electron supply for efficient catalysis of P450 systems. Finally, tryptophan‐5‐hydroxylase (T5H) activity (*K*_cat_ */K*_M_) in the optimal mutant is 36.62‐fold than that of wild‐type. The engineered strain *E. coli* S11 can produce 15.42 g L^−1^ serotonin in a 7.5‐L bioreactor, which is 9.17‐fold of the previous reported. This strategy provides a systematic approach for regulating ETF in complex P450 catalytic systems for efficient chemical biosynthesis.

## Graphical abstract

The electron transfer flux in CPR‐P450 catalytic system is systematically engineered through: i) enhancing electron transfer rate by redesigning the putative electron transfer pathway of CPR; ii) improving electron‐receiving rate by evolving the heme domain of tryptophan‐5‐hydroxylase (T5H); iii) enlarging electron supply by fine‐tuning NADPH synthesis. This significantly enhances the activity of T5H and the serotonin production in *Escherichia coli*.

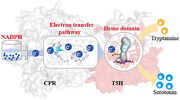

## 1 Introduction

Cytochrome P450 enzymes (P450s) can catalyze various reactions such as hydroxylation, C═C epoxidation, N‐ or S‐oxidation/dealkylation, and C─C cleavage by using a heme group to introduce an oxygen atom into an inactive C─H bond,^[^ ^1^ ^]^ which makes them powerful tools for building cell factories to produce high‐value natural products, including terpenoids,^[^ ^2^ ^]^ fatty acids,^[^ ^3^ ^]^ amino acid derivatives,^[^ ^4^ ^]^ among others.^[^ ^5^ ^]^ However, the activity of recombinantly expressed P450 enzymes in whole cells is relatively weak because of factors such as electron transfer,^[^ ^6^ ^]^ heme supply,^[^ ^7^ ^]^ and oxygen supply^[^ ^8^ ^]^ that hinder the efficient synthesis of natural products. For example, tryptophan‐5‐hydroxylase (T5H), a member of the P450s, can catalyze the hydroxylation of tryptophan to produce serotonin, an important amino acid derivative, medicinal neurotransmitter and functional supplement.^[^ ^9^ ^]^ Heterologous expression of T5H (from *Oryza sativa*) in *Escherichia coli* has weak catalytic activity, resulting in a low serotonin yield of only 250 mg L^−1^ (Table S1, Supporting Information). This cannot meet the requirements of industrial production.^[^ ^10^ ^]^

Protein engineering based on rational design or directed evolution has been used to generate mutants with improved characteristics to enhance P450 catalytic activity.^[^ ^11^ ^]^ However, effective and expeditious selection of beneficial variants with the desired phenotype from a large mutation library remains a challenge. In this case, a biosensor that can rapidly monitor small intracellular molecules in real time is suitable for studying the evolution of enzymes and strains, metabolic regulation, and the visualization and detection of compounds.^[^ ^12^ ^]^ Especially, genetically encoded biosensors such as transcription factor‐based biosensors, DNA biosensors and riboswitches, which have high specificity and sensitivity for target metabolites, can convert inconspicuous metabolites into measurable signals or link the characteristics of enzymes to cell survival at the single‐cell level, enabling highly accurate cell analysis and screening.^[^ ^13^ ^]^ Thus, a genetically encoded biosensor that specifically responds to serotonin is essential for rapidly screening highly active T5H mutants. Recently, two RNA aptamers capable of binding serotonin have been developed through in vitro selection.^[^ ^14^ ^]^ However, developing an RNA aptamer into a genetically encoded biosensor to detect intracellular metabolite concentrations in real time remains challenging. Stojanovic et al.^[^ ^15^ ^]^ reported a modular aptamer sensor strategy for designing an RNA biosensor that could monitor and track the distribution and dynamics of metabolites. This modular aptameric sensor‐comprising a reporting domain, recognition domain and communication module‐is capable of transducing recognition events into fluorescence changes through the allosteric regulation of non‐covalent interactions with a fluorophore.^[^ ^16^ ^]^ The design of modular aptameric sensors is highly universal because the recognition domain can be functionally exchanged for any predefined or identified RNA aptamer.^[^ ^12a^ ^]^ Thus, the construction of modular aptamer sensors using serotonin RNA aptamers as recognition domains could aid in the rapid development of novel genetically encoded serotonin RNA biosensors.

A series of mutation sites were predicted using enzyme structure, catalytic mechanism and computational technology. This enabled us to obtain highly active mutants more easily. The catalytic mechanism of P450s requires two electrons provided by NAD(P)H, which are ultimately transferred to heme.^[^ ^1a^ ^]^ Electron transfer is regarded as the rate‐limiting step in the synthesis of natural products catalyzed by P450s.^[^ ^8^, ^17^ ^]^ Two strategies have been reported to increase the electron transfer flux (ETF) to provide the electron supply required for P450 catalysis. i) Increasing the accumulation of the electron donor NADPH, such as overexpression of the glucose dehydrogenase for NADPH synthesis.^[^ ^18^ ^]^ ii) Engineering electron transfer pathways (ETPs). This strategy mainly focuses on P450 BM3, which is classified as a rare one‐component P450 catalytic system that includes a heme domain and a reductase domain.^[^ ^19^ ^]^ However, these strategies are still limited in improving the yield of P450‐catalyzed natural products, possibly because they consider only a single factor that regulates the ETF, ignoring the overall processes of electron transfer and cannot systematically regulate the ETF in a multi‐pronged manner. Furthermore, most P450s require one or two redox partners to form two‐ or three‐component P450 catalytic systems, in which redox partners are considered to be auxiliary electron transfer proteins.^[^ ^20^ ^]^ In three‐component P450 catalytic systems, the redox partners are flavin adenine dinucleotide (FAD)‐containing ferredoxin reductase and iron‐sulfur‐containing ferredoxin. In two‐component P450 catalytic systems, the redox partner, also called P450 reductase (CPR), incorporates two cofactors, FAD and Flavin Mononucleotide (FMN). The CPR is directly coupled to NAD(P)H to acquire electrons that are subsequently transferred to the heme domain in P450. Ultimately, electrons are received by heme to reduce oxygen and facilitate hydroxylation of the substrate.^[^ ^17^ ^]^ For example, T5H requires CPR to form a two‐component P450 catalytic system, and the heterologous co‐expression of T5H and CPR can significantly increase serotonin production (Table S1, Supporting Information).^[^ ^10^ ^]^ Therefore, systematic engineering of electron transfer is necessary to increase the ETF, thereby enhancing P450 activity in two‐ or three‐component P450 catalytic systems.

In this study, we developed a systematic evolutionary engineering method to increase ETF in a two‐component P450 catalytic system using T5H and CPR as a model system. Based on the entire electron transfer process, the ETF was engineered from three perspectives: the electron transfer rate, electron acceptance rate, and electron donor availability. A novel genetically encoded serotonin RNA biosensor was constructed and coupled with virtual computing to recognize and evolve putative ETP in the CPR and enhance the electron transfer rate. Next, the heme‐binding domain in T5H was determined using molecular docking and evolved to improve the electron‐receiving rate. Furthermore, the availability of the electron donor NADPH was improved by fine‐tuning the NADPH metabolism. Finally, mutants for engineered ETF were used to construct cell factories for efficient serotonin synthesis in *E. coli*. Overall, our work represents a novel and universal approach to engineering ETF to enhance the catalytic activity of P450 in two‐component P450 catalytic systems.

## 2 Results

### 2.1 Developing an RNA‐Biosensor for High‐Throughput Screening of Intracellular Serotonin

**Figure 1:**
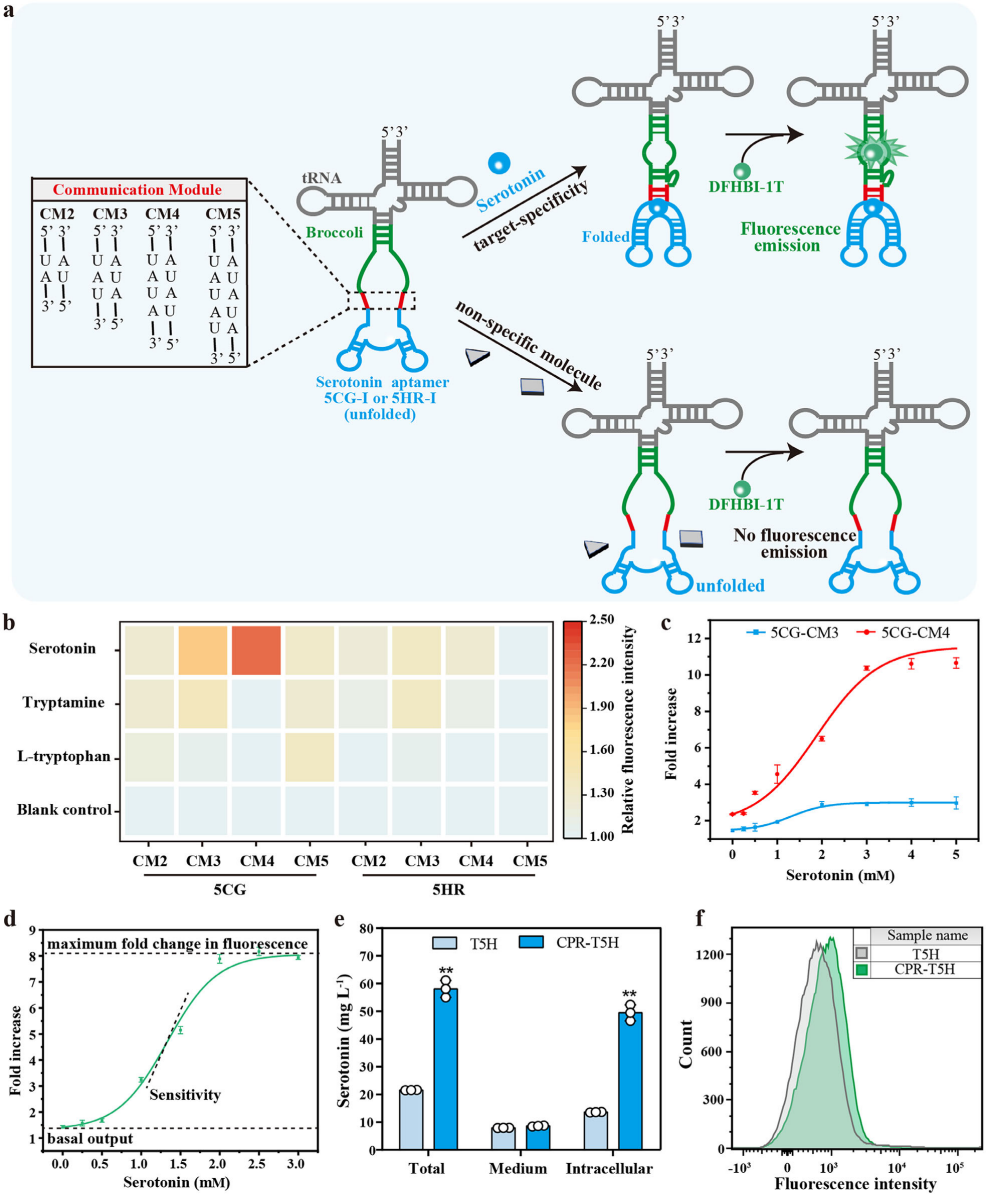
Screening and optimization of serotonin RNA‐biosensor for electronic transfer engineering. a) Schematic representation of the secondary structure of a gene‐encodable serotonin biosensor. b) Heat map describing the specificity of biosensors constructed by 5CG/5HR‐I aptamer and different CMs in vivo. c) Dose‐response plots of 5CG‐CM3 and 5CG‐CM4 biosensors *in E.coli*. d) Dose‐response plots of 5CG‐CM4 biosensor in vitro. Curves represent the fit for the Boltzmann equation. e) The serotonin production in CPR‐T5H and T5H strains under whole‐cell catalytic reaction for 3h. f) Fluorescence intensity at the single‐cell level was measured by FACS under whole‐cell catalytic reaction for 3h. The error bars indicate the standard deviation of three biological replicates (n = 3). Significance was analyzed using a t‐test. Statistical significance is indicated as * for *P* < 0.05 and ** for *P* < 0.01, respectively.

A novel genetically‐encodable serotonin RNA biosensor was constructed in vivo using a modular aptamer sensor construction method^[^ ^15^ ^]^ to detect serotonin production in *E. coli* and screen for highly active mutants in the high‐complexity library of evolutionary engineering of ETF. As shown in **Figure** **1**a, the serotonin RNA biosensor contained four modules: tRNA scaffold, fluorescent aptamer ‘Broccoli’ (reporting domain), communication module (CM), and serotonin RNA aptamer (recognition domain), in which the tRNA scaffold stabilizes the biosensor for cell‐based applications.^[^ ^21^ ^]^ The serotonin aptamer undergoes ligand‐induced structural reorganization upon binding serotonin, forming a tight (folded) conformation. This conformational transition was transmitted to the fluorescent aptamer domain via the CM, triggering its structural shift to a folded conformation. This facilitated the binding of the fluorescent dye DFHBI‐1T to the fluorescent aptamer and achieved fluorescence emission. Conversely, in the absence of serotonin, both the serotonin and fluorescent aptamers maintained their unfolded conformations, DFHBI‐1T could not bind to the fluorescent aptamer, and fluorescence was not generated. Notably, the CM is responsible for connecting the serotonin RNA aptamer and the fluorescent RNA aptamer and is critical in the signal conversion between the serotonin concentration and the fluorescence intensity. The GC content of the biosensor was ≈60.23% except for the CM module. Introducing of the U‐A combination into the CM module can reduce the overall GC content, reducing the structural rigidity and facilitating conformational changes and transmission. Thus, CM modules with different lengths of U‐A combinations were selected for coupling serotonin‐responsive 5CG‐I or 5HR‐I^[^ ^14^ ^]^ and fluorescent aptamers to construct biosensors. Eight RNA biosensors were generated, and their in vivo specificities for L‐tryptophan, tryptamine, and serotonin were evaluated separately (Figure 1b). The 5CG‐CM4 biosensor exhibited a specific response to serotonin. The relative fluorescence intensity of 5CG‐CM4 after serotonin induction was 2.21‐fold than that of the blank control. In contrast, the relative fluorescence intensity of 5CG‐CM4 after L‐tryptophan and tryptamine induction was did not differ significantly from that of the blank control. The 5CG‐CM3 biosensor responded to serotonin and tryptamine with relative fluorescence intensities of 1.8‐fold and 1.42‐fold, respectively, compared to those of the blank control upon serotonin and tryptamine induction. The relative fluorescence intensity of 5CG‐CM3 after tryptamine induction did not differ significantly from that of the blank control. The remaining six biosensors did not specifically respond to serotonin. Next, the dose‐response curves of the 5CG‐CM3 and 5CG‐CM4 biosensors in response to serotonin were obtained. As shown in Figure 1c, the 5CG‐CM4 biosensor operated within the 0.25‐3 mm serotonin, achieving a maximum specific fluorescence fold increase of 10.85‐fold. Conversely, the 5CG‐CM3 biosensor functioned within a narrower range of 0.25–2 mm serotonin, with a maximum specific fluorescence fold increase of only threefold. The 5CG‐CM4 biosensor responded to a broader range of serotonin concentrations and exhibited superior fluorescence intensity compared to the 5CG‐CM3. We also investigated the in vitro performance of the 5CG‐CM4 biosensor. As shown in Figure 1d, the 5CG‐CM4 biosensor in vitro operated in the range of 0.5–2 mm serotonin, achieving a 7.94‐fold maximum fold increase in fluorescence. 5CG‐CM4 was more sensitive to serotonin under in vitro than under in vivo, however it responded to a narrower linear range of serotonin, with a smaller maximal signal amplitude and lower basal output (Table S2, Supporting Information). We performed recovery experiments both in vivo and in vitro systems using high‐performance liquid chromatography (HPLC) as the reference method to assess the quantitative accuracy. The recovery of serotonin was 116% using the biosensor and 100% using the HPLC assay in the in vivo system (Table S3, Supporting Information). The recovery of serotonin was 105.33% using the biosensor and 100% using the HPLC assay in the in vitro system. These results indicated that the biosensor 5CG‐CM4 could be used for rapid quantitative serotonin detection in vivo and in vitro.

Two strains, CPR‐T5H and T5H, were constructed with using the 5CG‐CM4 biosensor to demonstrate the feasibility of the serotonin biosensor for engineering ETF in vivo. CPR is responsible for the obtaining electrons from NADPH during the synthesis of serotonin using T5H. Subsequently, the enzyme kinetic parameters of T5H and CPR‐T5H were determined. In the pure‐enzyme system (Figure S1, Supporting Information), T5H alone did not catalyze serotonin synthesis, which was catalyzed by both CPR and T5H (**Table** **1**). To assay the enzyme activities of T5H and CPR‐T5H during the whole‐cell catalytic stages, *V*_max_ levels of the lysates after normalizing the total protein content were measured to assay the enzyme activities of T5H and CPR‐T5H during the whole‐cell catalytic stages. The value of *V*_max_ for CPR‐T5H was significantly higher than that of T5H. This suggested that the co‐expression of CPR with T5H significantly enhanced the catalytic activity of T5H. Additionally, the whole‐cell‐catalyzed serotonin yield in the CPR‐T5H strain was 175.12 mg L^−1^, which was 2.89‐fold higher than that of T5H (60.61 mg L^−1^) (Figure S2a, Supporting Information). However, there was no significant difference in fluorescence intensity between the CPR‐T5H and T5H strains at the single‐cell level as measured using flow cytometry (Figure S2b, Supporting Information). Further detection of intracellular and extracellular serotonin content showed that most serotonin was secreted into the culture medium, resulting in no significant difference in intracellular serotonin content between the CPR‐T5H and T5H strains (Figure S2a, Supporting Information). The diffusion of serotonin could interfere with the binding and fluorescence responses of the 5CG‐CM4 biosensor to intracellular serotonin. Additionally, serotonin presented in the medium could re‐enter the cell and bind to the biosensor, generating strong fluorescence and causing crosstalk during fluorescence‐activated cell sorting (FACS). To address this issue, we minimized the diffusion of serotonin by performing a protein induction reaction and enzyme catalysis in stages and optimizing the catalytic time, given that enzyme catalysis and serotonin diffusion were sequential in the preliminary phase (Figure S2c, Supporting Information). Under whole‐cell catalytic reaction for 3h, the intracellular serotonin content in the CPR‐T5H strain (49.49 mg L^−1^) was significantly higher than that of T5H strain (13.61 mg L^−1^), and only a small amount of serotonin accumulated in the culture medium (Figure 1e). The difference in fluorescence between the CPR‐T5H and T5H strains was maximal when T5H was catalyzed for 3 h (Figure 1f; Figure S2d–f, Supporting Information). In summary, a novel genetically encoded serotonin biosensor was constructed using a modular aptamer sensor strategy that can be used for high‐throughput screening of intracellular serotonin.

**Table 1:** Enzyme kinetic parameters for T5H and CPR‐T5H.

		*K*_M_ [mM]	*V*_max_ [µM min^−1^]	*K*_cat_ [min^−1^]
In the pure enzyme systems	T5H^a^ ^)^	‐^b)^	–	–
	CPR‐T5H^c)^	1.56±0.80	0.20±0.05	0.20±0.05
In cell lysates	T5H	2.25±1.03	0.03±0.01	–
	CPR‐T5H	1.25±0.02	0.42±0.01	–

^a)^ The reaction system of T5H contained 1 µm purified T5H, 5 mm NADPH, 0.5 to 3 mm tryptamine, and Tirs‐Hcl buffer (50 mm, pH 7.5) in a total volume of 500 µL;

^b)^ “‐” indicated not detected;

^c)^ The reaction system of CPR‐T5H contained 1 µm purified CPR (105 residues at the N‐terminus of the purified CPR enzyme were deleted), 1 µm purified T5H, 5 mm NADPH, 0.5 to 3 mm tryptamine, and Tirs‐Hcl buffer (50 mm, pH 7.5) in a total volume of 500 µL.

### 2.2 Enhancing Electron Transfer Rate by Redesigning the Putative Electron Transfer Pathway of CPR

**Figure 2:**
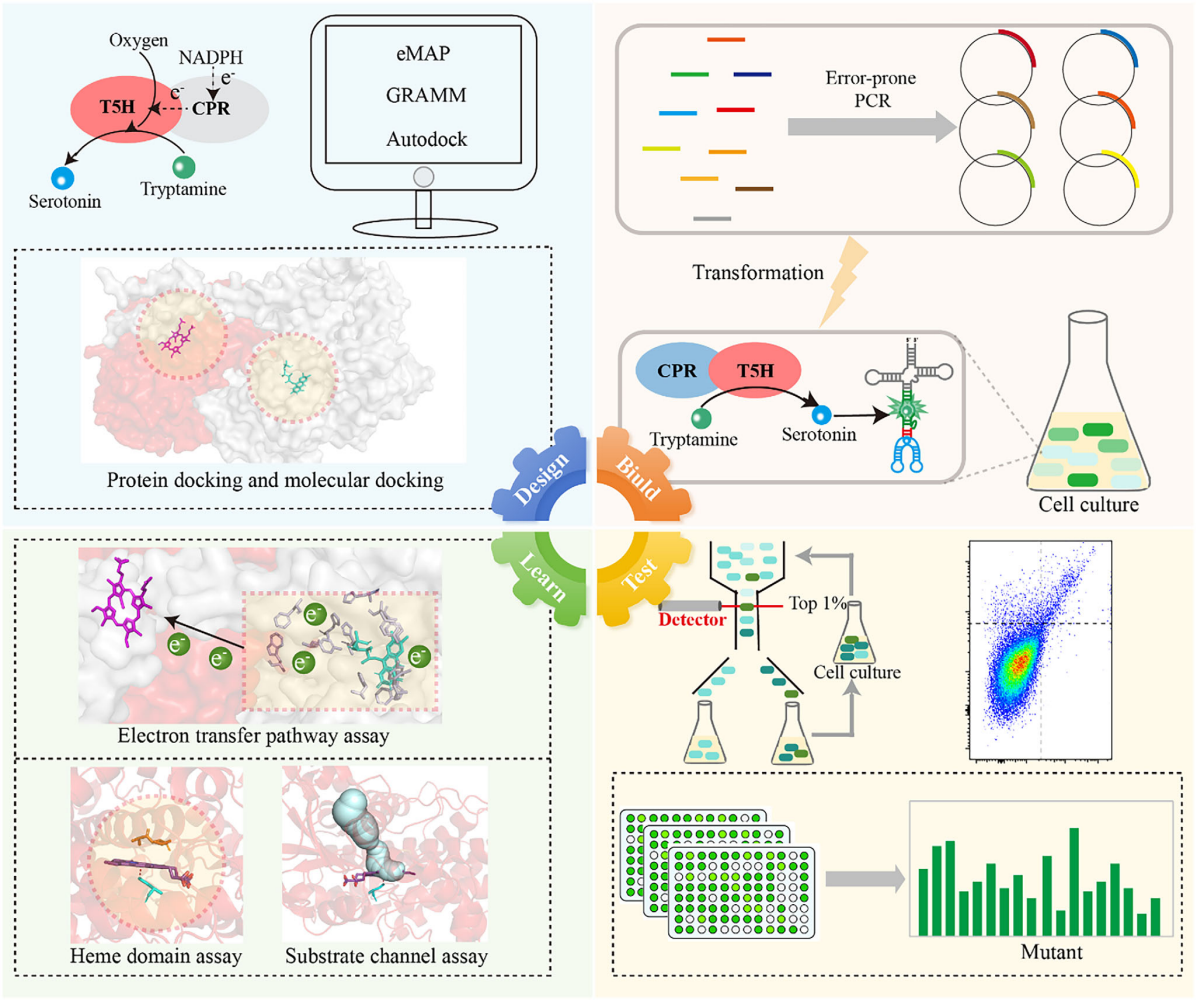
A DBTL framework of coupled serotonin RNA biosensors for ETF engineering. “Design”: Identification of the critical regions affecting electron transfer flux through virtual computing websites and softwares such as eMAP, Gramm and Autodock. “Build”: Construction of the mutants with serotonin biosensor. “Test”: Screening for mutant strains with high‐production of serotonin. “Learn”: Analyzing the mechanism of high catalytic activity of enzyme mutants.

A serotonin RNA biosensor coupled to a design‐build‐test‐learn (DBTL) framework was used to identify and evolve critical regions affecting ETF and investigate the effect of ETF on enzyme activity. As shown in **Figure** **2**, the regions affecting ETF in T5H and CPR were identified using virtual computing. Subsequently, random mutant libraries targeting these key regions were constructed using error‐prone PCR. Next, the mutant libraries were subjected to high‐throughput screening to identify the mutants with enhanced activity. Finally, the molecular mechanisms by which the mutants affect ETF were analyzed.

#### 2.2.1 Identifying the Electron Transfer Pathways in CPR

**Figure 3:**
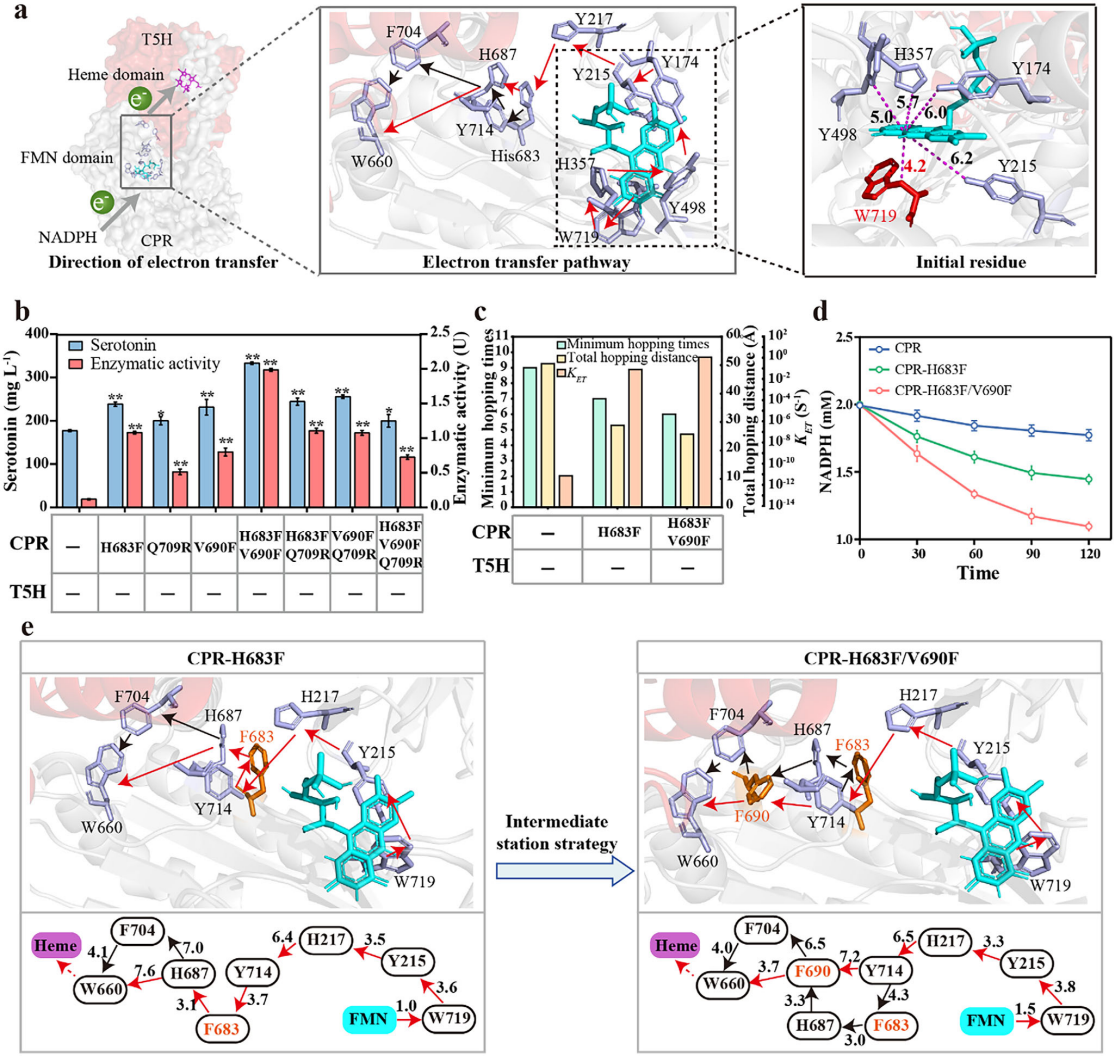
Re‐design of the putative electron transfer pathway (ETP) in CPR to improve electron transfer rate. a) Identification of the possible ETPs in CPR. b) The production of serotonin and enzyme activities of the CPR mutants. 1 U is defined as the amount of enzyme required to catalyze the production of serotonin from 1 µm tryptamine per minute. The reaction system of CPR‐T5H (or their mutants) contained 1 µm purified CPR or CPR mutants (105 residues at the N‐terminus of the purified CPR proteins were deleted), 1 µm purified T5H, 5 mm NADPH, 3 mm tryptamine, and Tirs‐Hcl buffer (50 mm, pH 7.5) in a total volume of 500 µL. “‐” indicated no mutant. c) The calculation of electron transfer rate of the CPR mutants. d) After normalizing the total protein content, the rate of NADPH consumption in cell lysates. e) eMAP predicted the ETP of CPR‐H683F and CPR‐H683F/V690F. The shortest electron transfer directions (red arrows), the branching path of electron transfer directions (black arrows). The error bars indicate the standard deviation of three biological replicates (n = 3).Significance was analyzed using a t‐test. Statistical significance is indicated as * for *P* < 0.05 and ** for *P* < 0.01, respectively.

In the two‐component P450 catalytic system, the electron donor NADPH enters the FAD domain of CPR, FAD gains two electrons and transfers them sequentially to FMN. Next, the reduced FMN sequentially delivers two electrons to the P450 enzyme via the ETP in the CPR.^[^ ^22^ ^]^ Thus, we first investigated the ETP in CPR (*Oryza sativa*) to enhance the electron transfer rate in CPR. In general, amino acids containing aromatic side chains and aromatic fragments (tryptophan, tyrosine, phenylalanine and histidine) are considered potential participants in electron transfer.^[^ ^23^ ^]^ The ETP of the CPR was predicted using eMAP, which revealed a variety of residues that might be involved in electron transport. (Figure S3, Supporting Information). The docking structure of CPR with FMN was generated to identify the initiating residue of ETP (**Figure** **3**a). The residues involved in the ETP around the FMN included Y174, Y215, H357, Y498, and W719, with W719 being the closest to the C‐atom that receives electrons in the FMN (the C‐atom located between C4 and N5 in isoalloxazine). Thus, W719 was confirmed to be the initial residue of ETP in the CPR. We generated protein docking structures for CPR and T5H using GRAMM^[^ ^24^ ^]^ (Figure 3a) to determine the orientation of ETP. This showed that the terminal residue of ETP in the CPR might be W660. These results revealed that the electrons in the CPR may be transferred from FMN to W660, with intermediate residues involved in ETP including W719, H357, Y489, Y174, Y215, H217, H683, Y714, H687, and F704.

#### 2.2.2 Evolving the Putative Electron Transfer Pathways in CPR

Second, a random mutation library targeting residues involved in the putative ETP and their surrounding regions (Residues 170–220, 350–490, and 680–714) was generated using error‐prone PCR in CPR‐T5H (wild‐type, WT) and the 5CG‐CM4 biosensor. A multistep FACS enrichment strategy was employed, and highly fluorescent cells were progressively enriched after three rounds of sorting (Figure S4a, Supporting Information). The top 1% of the fluorescent cells were collected and transferred to 96‐well plates for single‐cell culture, and their fluorescence intensities were measured using a multifunctional enzyme marker. Sixteen mutants exhibited superior fluorescence output and were further inoculated into shake flasks for whole‐cell catalysis and assayed for serotonin production (Figure S4b, Supporting Information). Two mutants, CPRM5‐T5H (with the mutation site identified as CPR‐H683F‐T5H via Sanger sequencing) and CPRM14‐T5H (CPR‐Q709R‐T5H) had serotonin yields of 243.34 and 209.69 mg L^−1^, respectively (Figure 3b), representing increases of ≈38.95% and 19.73%, respectively, compared to that of the WT (175.12 mg L^−1^). Subsequently, the enzyme kinetic parameters of CPR‐H683F‐T5H and CPR‐Q709R‐T5H were determined. The *K*_cat_ */K*_M_ values of CPR‐H683F‐T5H and CPR‐Q709R‐T5H were 1.57 and 0.58 mm ^−1^ min^−1^, respectively, which were 12.08 and 4.46 folds that of WT (0.13 mm ^−1^ min^−1^), respectively (**Table** **2**). The *K*_M_ values of CPR‐H683F‐T5H was 0.99±0.41 mm tryptamine, which was significantly lower than that of the WT (1.56±0.80 mm tryptamine). The *K*_M_ values of CPR‐Q709R‐T5H was 1.44±0.73 mm tryptamine, which did not differ significantly from that of WT (1.25±0.02 mm tryptamine). These results indicated that the catalytic activity of T5H was significantly improved in CPR‐H683F‐T5H and CPR‐Q709R‐T5H. Additionally, the affinity of T5H for tryptamine was also increased in CPR‐H683F‐T5H.

**Table 2:** Enzyme kinetic parameters for the CPR and T5H mutants.

	*K*_M_ [mm]^a)^, ^b)^	*K*_cat_ [min^−1^]	*K*_cat_ */K*_M_ [mm ^−1^ min^−1^]
CPR‐T5H (WT)	1.56±0.80	0.20±0.05	0.13
CPR‐H683F‐T5H	0.99±0.41	1.55±0.25	1.57
CPR‐Q709R‐T5H	1.44±0.73	0.83±0.19	0.58
CPR‐H683F/V690F‐T5H (mCPR‐T5H)	1.01±0.59	2.90±0.65	2.87
mCPR‐T5H‐T331C/V338L	1.04±0.59	3.74±0.83	3.60
mCPR‐T5H‐A337T/V338L	1.09±0.60	3.48±0.75	3.19
mCPR‐T5H‐T331C/A337F (mCPR‐mT5H)	0.93±0.39	4.43±0.69	4.76

^a)^ 105 residues at the N‐terminus of the purified CPR and CPR mutants were deleted;

^b)^ The reaction system of CPR‐T5H (or their mutants) contained 1 µm purified CPR (or CPR mutants), 1 µm purified T5H (or T5H mutants), 5 mm NADPH, 0.5 to 3 mm tryptamine, and Tirs‐Hcl buffer (50 mm, pH 7.5) in a total volume of 500 µL.

The 3D structures and ETPs of CPR‐H683F and CPR‐Q709R were analyzed using αFold2 and eMAP, respectively, to explore the effect of mutants on electron transfer. The results showed no significant change in the residues of the putative ETP in CPR‐Q709R (Figure S4c, Supporting Information). Molecular dynamics simulations showed that an increase in the root mean square fluctuation (RMSF) at the C‐terminus of CPR‐Q709R might enable the residues involved in electron transfer to become more flexible, which in turn would improve the electron transfer rate and T5H catalytic activity (Figure S4d,e, Supporting Information). Notably, the electrons were transferred directly to Y215 via W719 in CPR‐H683F without passing through H357, Y489, or Y174 (Figure 3e). The transfer processes of electrons from FMN to W660 in CPR‐H683F required only to hop seven times, and the shortest distance crossed was 28.9 Å. However, the electrons in wild CPR were required to hop nine times, and the shortest distance crossed was 50.8 Å (Figure 3c; Figure S4f, Supporting Information). The calculations of electron transfer rate (*Κ*_ET_) showed that the *Κ*_ET_ from FMN to W660 was only 9.93 × 10^−2^ S^−1^ in CPR‐H683F (Figure 3c), whereas the *Κ*_ET_ was 8.23 × 10^−12^ S^−1^ in wild CPR. These results indicated that the mutant CPR‐H683F had a higher electron‐hopping rate than the wild‐type CPR. After normalizing the total protein content, the rate of NADPH consumption was assayed in the cell lysate was assayed to evaluate the electron transfer rate. CPR‐H683F‐T5H consumed a total of 0.55 mm NADPH after 120 min of reaction, while only 0.22 mm NADPH was consumed in the WT (Figure 3d, Supporting Information). This result showed that the amount of NADPH consumed by CPR‐H683F was 2.50‐fold compared to that of the WT during the same catalytic time. Consequently, we found that the electron transfer rate could be increased by shortening the number of steps and distances of electron multistep hopping in the putative ETP of CPR, thereby enhancing T5H catalytic activity and the affinity of T5H for tryptamine.

#### 2.2.3 Redesigning the Electron Transfer Pathway in CPR

Third, an intermediate station strategy was used to reduce the number of steps and distance of electron multistep hopping to further enhance the catalytic activity of T5H. Electrons were transferred via a curvilinear route between Y714 and W660, therefore, aromatic amino acids or histidine were introduced into the residues between Y714 and W660 (Figure S4g, Supporting Information), which acted as intermediate sites for electron transfer. Subsequently, CPR‐H683F/V690F was identified using eMAP. The transfer processes of electrons from W719 to W660 in CPR‐H683F/V690F required only to hop 6 times, and the shortest distance crossed was 26 Å, resulting in *Κ*_ET_ of 2.15 S^−1^ (Figure 3c). Besides, CPR‐H683F/690F‐T5H consumed 0.90 mm NADPH after 120 min of reaction (Figure 3d). The amount of NADPH consumed by CPR‐H683F/690F‐T5H was 4.09‐fold compared to that of WT. These results suggested that establishing an intermediate station for electron transfer in CPR‐H683F/V690F‐T5H further enhanced the electron transfer rate compared with CPR‐H683F‐T5H. Additionally, the serotonin yield of CPR‐H683F/V690F‐T5H was 335.78 mg L^−1^ (Figure 3b), which was ≈91.74% higher than that of the WT. We constructed double‐ and triple‐point mutants based on three single‐point mutants, CPR‐H683F, CPR‐Q709R, and CPR‐V690F, and screened the optimal mutants using whole‐cell catalytic activity and enzyme activity assays to systematically evaluate the catalytic activity of CPR mutants. As shown in Figure 3b, whole‐cell catalyzed serotonin production and enzyme activity of CPR‐H683F/V690F‐T5H were significantly higher than those of the other mutants. The enzyme kinetic parameters of CPR‐H683F/V690F‐T5H were determined. The value of *K*_cat_ */K*_M_ for CPR‐H683F/V690F‐T5H was 2.87 mm ^−1^ min^−1^, which was ≈22.08 folds than that of WT. The *K*_M_ values of CPR‐H683F/V690F‐T5H was 1.01±0.59 mm tryptamine, which was significantly lower than that of WT. This indicated that the catalytic activity of T5H and the production of serotonin were further improved in CPR‐H683F/V690F.

In contrast, CPR‐H683F/Y215A‐T5H and CPR‐H683F/H217A‐T5H showed a disconnection of the putative ETP or a substantial increase in the number of residues (Figure S5a,b, Supporting Information). They exhibited serotonin yields of 109.10 and 129.22 mg L^−1^, respectively, representing reductions of ≈37.70% and 26.21% compared to WT (Figure S5c, Supporting Information). The enzyme activities of T5H in CPR‐H683F/Y215A‐T5H and CPR‐H683F/H217A‐T5H were 0.07 and 0.09 U, respectively, which were significantly lower than that of CPR‐H683F (1.08 U) and also slightly reduced compared with WT (0.12 U). Subsequently, considering that the iron ions in heme would present a sequential change in Fe^III^, Fe^II^, and Fe^III^ before and after receiving two electrons, the relative change levels of Fe^II^ before and after the enzyme‐catalyzed reaction were detected to characterize the ETF in CPR‐T5H‐catalyzed cycles. Before T5H catalyzed the reaction, the relative levels of Fe^II^ in the CPR mutants showed no significant changes compared with those in the WT (Figure S5d, Supporting Information). After 15 min of the T5H‐catalyzed reaction, the content of Fe^II^ in CPR‐H683F‐T5H (1.39) and CPR‐H683F/V690F‐T5H (1.63) was significantly higher than that in the WT; whereas the content of Fe^II^ in CPR‐H683F/Y215A‐T5H (1.03) and CPR‐H683F/H217A‐T5H (1.07) was significantly lower than that in CPR‐H683F‐T5H and slightly lower than that in the WT (1.09). The above results indicated that the putative ETP in the CPR significantly affected the ETF in CPR‐T5H catalyzed reaction, significantly influencing the T5H catalytic activity.

In summary, evolutionary engineering and intermediate station strategies were performed to shorten electron‐hopping steps and distance of the putative ETP of CPR. This facilitated the enhancement of the electron transfer rate and resulted in the improvement of T5H catalytic activity and T5H affinity for the substrate tryptamine.

### 2.3 Improving Electron Receiving Rate by Evolving the Heme Domain of T5H

**Figure 4:**
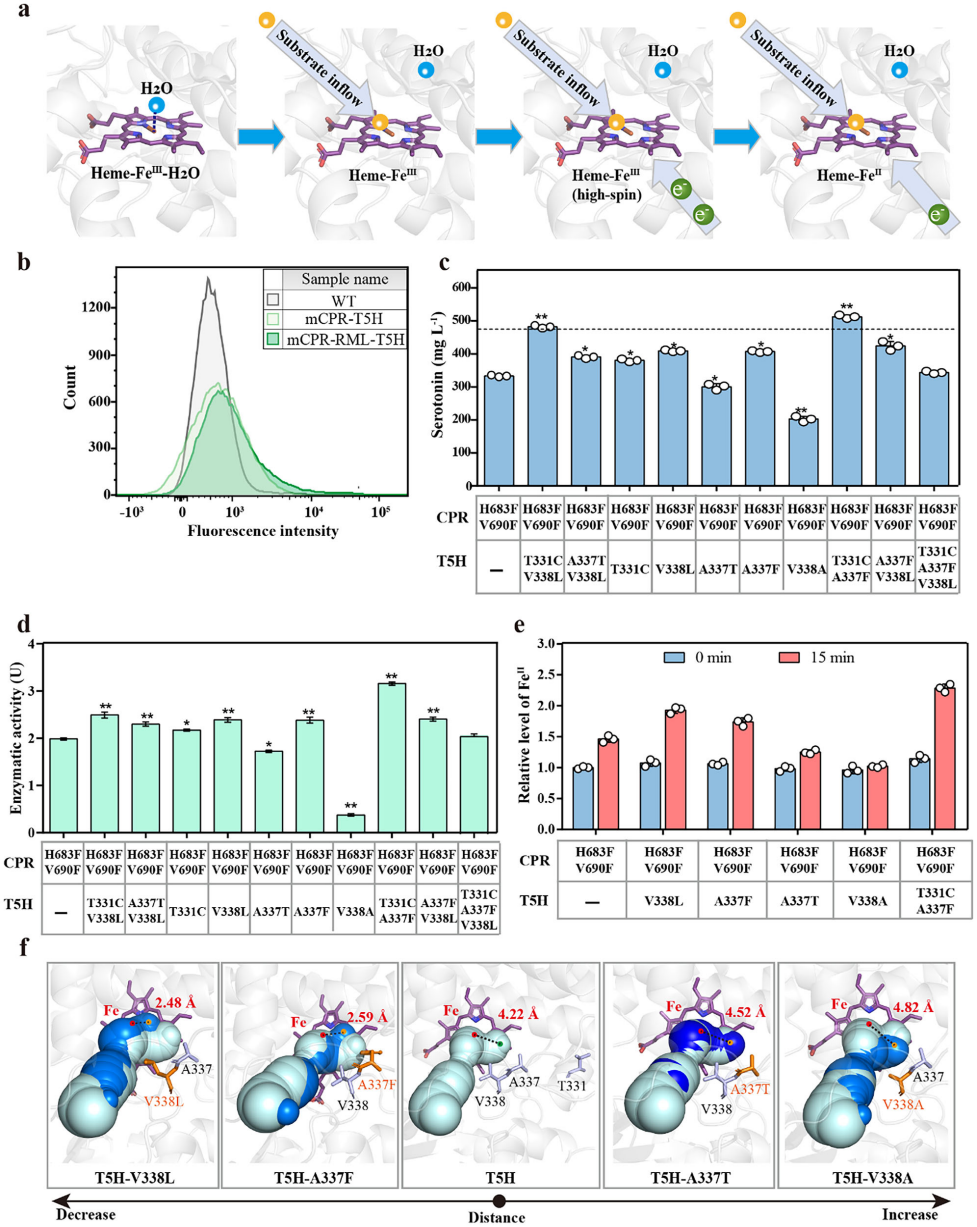
Screening and designing of highly active T5H mutants via evolving the heme domain of T5H. a) Schematic representation of the electron receiving process in heme domain of P450. Substrate molecule (yellow spheres), water molecule (blue spheres). b) Random mutation library of T5H (mCPR‐RML‐T5H) with higher fluorescence intensity at the single‐cell level using biosensor‐based FACS‐screening. c) The serotonin production of the T5H mutants with the co‐expression of mCPR (CPR‐H683F/V690F). “‐” indicated no mutant. d) The enzyme activities of the T5H mutants. 1 U was defined as the amount of enzyme required to catalyze the production of serotonin from 1 µm tryptamine per minute. The reaction system of CPR‐T5H (or their mutants) contained 1 µm purified mCPR (105 residues at the N‐terminus of the purified mCPR proteins were deleted), 1 µm purified T5H or CPR mutants, 5 mm NADPH, 3 mm tryptamine, and Tirs‐Hcl buffer (50 mm, pH 7.5) in a total volume of 500 µL. “‐” indicated no mutant. e) The relative levels of Fe^II^ in cell lysates before and after 15 min of the catalytic reaction. f) The distances between the terminal points of the substrate channel and heme‐Fe were measured. Heme‐Fe point (red circle), the terminal point of the substrate channel in T5H (green circle), the substrate channel in T5H (pale cyan) and T5H mutants (marine). The error bars indicate the standard deviation of three biological replicates (n = 3). Significance was analyzed using a t‐test. Statistical significance is indicated as * for *P* < 0.05 and ** for *P* < 0.01, respectively.

The heme in P450 is the final receptor for electron transfer. During the electron‐receiving process, the bound water molecule from resting‐state heme‐Fe^III^‐OH_2_ was displaced by the substrate to generate high‐spin Fe^III^. The high‐spin Fe^III^ then receives one electron from CPR and generates Fe^II^, thereby initiating oxygen reduction (**Figure** **4**a).^[^ ^5^, ^20^ ^]^ The heme‐binding domain in T5H, where Heme‐Fe was linked to the sulfur group of C416 to form an axial ligand, was identified using the docking structure of heme and T5H to improve the ETF during electron reception further (Figure S6a, Supporting Information). A random mutant library of residues in the heme‐binding domain (residues L59‐L106, F241‐R344 and P408‐V426, Figure S6b, Supporting Information) was constructed using error‐prone PCR in mCPR‐T5H (CPR‐H683F/V690F‐T5H). Similarly, the 5CG‐CM4 biosensor was combined with FACS for the high‐throughput screening of highly active T5H mutants (Figure 4b). Sixteen mutants exhibiting superior fluorescence output were identified using rescreening with a multifunctional enzyme marker (Figure S6c, Supporting Information). Two mutants exhibiting increased serotonin production, mCPR‐T5H‐M7 (with the mutation site determined as mCPR‐T5H‐T331C/V338L) and mCPR‐T5H‐M10 (mCPR‐T5H‐A337T/V338L), were identified using HPLC, with the serotonin yields of 486.14 mg L^−1^and 395.45 mg L^−1^, respectively (Figure 4c), which were ≈44.78% and 17.77% higher than that of the mCPR‐T5H. The enzyme kinetic parameters of mCPR‐T5H‐T331C/V338L and mCPR‐T5H‐A337T/V338L were measured. The *K*_cat_ */K*_M_ values of mCPR‐T5H‐T331C/V338L and mCPR‐T5H‐A337T/V338L were 3.60 and 3.19 mm ^−1^ min^−1^, respectively, which were 1.25 and 1.11 folds of that of mCPR‐T5H, respectively (Table 2). The *K*_M_ values of mCPR‐T5H‐T331C/V338L and mCPR‐T5H‐A337T/V338L were 1.04±0.59 mm tryptamine and 1.09±0.60 mm tryptamine, respectively, which were slightly increased compared to that of mCPR‐T5H (1.01±0.59 mm tryptamine). These results indicate that the evolution of the heme domain enhanced the T5H catalytic activity.

To investigate the effect of mutant residues in the heme‐binding domain on ETF, three single‐point mutants (mCPR‐T5H‐T331C, mCPR‐T5H‐V338L, and mCPR‐T5H‐A337T) were constructed. The serotonin yield of mCPR‐T5H‐T331C was 384.44 mg L^−1^, representing an increase of ≈14.49% compared to that with mCPR‐T5H (Figure 4c). As shown in Figure 4d, the enzymatic activity of T5H in mCPR‐T5H‐T331C was 2.19 U, which was significantly higher than that of mCPR‐T5H (1.98 U). The effect of the T5H‐T331C was analyzed using 100 ns molecular dynamics simulations (Figure S6d,e Supporting Information). Root mean square deviation (RMSD) analysis revealed that the overall stability of T5H‐T331C might have been enhanced. This could have contributed to the modest increase in the catalytic efficiency of T5H. The serotonin yields of mCPR‐T5H‐V338L and mCPR‐T5H‐A337T were 412.04 and 302.63 mg L^−1^, respectively, an increase of 22.71% and a decrease of 9.87%, respectively, compared with that of mCPR‐T5H (Figure 4c). The enzyme activities of T5H in mCPR‐T5H‐V338L and mCPR‐T5H‐A337T were 2.42 U and 1.72 U, which were higher in the former and lower in the latter compared with that of mCPR‐T5H (Figure 4d). Notably, residues V338 and A337 were close to the heme and were located at the end of the substrate‐access tunnel (Figure 4f). By comparing the substrate channel parameters of T5H‐V338L and T5H‐A337T, we found that there was no correlation between the channel length and channel radius‐both average and end‐of‐channel radius‐and T5H catalytic activity (Table S4, Supporting Information). A comparison of the substrate channels between these two single‐point mutants and wild T5H revealed that the terminal point of the substrate channels in T5H‐V338L (at a distance of 2.48 Å) was closer to heme‐Fe than that of wild T5H (4.22 Å). In contrast, the ends of the substrate channels in T5H‐A337T (4.52 Å) was farther from heme‐Fe (Figure 4f; Table S5, Supporting Information). Therefore, we suspected that a shorter distance between the end of the substrate channel and heme‐Fe might facilitate the displacement of the water molecule from heme‐Fe^III^‐OH_2_ by the substrate. This in turn would improve the electron‐receiving rate and catalytic activity of T5H.

To verify this hypothesis, two mutants T5H‐A337F and T5H‐V338A were constructed. The distance between the end of the substrate channel and heme‐Fe in T5H‐A337F was 2.59 Å, which was 1.63 Å decrease compared to T5H. In contrast, this distance for T5H‐V338A was 4.82 Å, representing an increase of 0.6 Å compared to T5H (Figure 4f; Table S5, Supporting Information). As expected, mCPR‐T5H‐A337F (409.62 mg L^−1^) showed a 21.98% increase in serotonin production compared to mCPR‐T5H, while mCPR‐T5H‐V338A (194.12 mg L^−1^) exhibited a 42.19% decrease in serotonin production (Figure 4c). The trends between the enzyme activities of mCPR‐T5H‐A337F (2.37 U) and mCPR‐T5H‐V338A (0.38 U) were consistent with those of whole‐cell‐catalyzed systems (Figure 4d). These results further suggested that the distance between the end of the substrate channel and the heme‐Fe can significantly affect the T5H catalytic activity, and the closer the distance, the higher the T5H catalytic activity. Subsequently, relative change levels of Fe^II^ before and after the enzyme‐catalyzed reaction were detected in the T5H mutants. Before T5H catalyzed the reaction, the relative levels of Fe^II^ in the mutants (mCPR‐T5H‐V338L, mCPR‐T5H‐A337F, mCPR‐T5H‐A337T and mCPR‐V338A) showed no significant changes compared with that in mCPR‐T5H (Figure 4e). After 15 min of catalysis reaction performed using T5H, the relative levels of Fe^II^ in mCPR‐T5H‐V338L and mCPR‐T5H‐A337F were 1.93 and 1.74, respectively, representing increases of ≈32.19% and 19.18% compared to that with mCPR‐T5H (1.46). Conversely, the relative levels of Fe^II^ in mCPR‐T5H‐A337T and mCPR‐V338A were 1.25 and 1.02, reflecting decreases of about 14.38% and 30.14%, respectively, relative to that in mCPR‐T5H, respectively. This indicated that the distance between the end of the substrate channel in T5H and heme‐Fe can significantly affect the electron‐receiving rate, thereby influencing T5H catalytic activity.

Finally, double and triple point mutants were constructed by combining T331C, A337F, and V338L to obtain a higher catalytic activity of T5H. Among these, mCPR‐T5H‐T331C/A337F exhibited the highest serotonin yield at 518.17 mg L^−1^, which was ≈54.32% higher than that of mCPR‐T5H (Figure 4c). The enzyme activity of T5H in mCPR‐T5H‐T331C/A337F was the highest at 3.16 U compared with T5H mutants. mCPR‐T5H‐T331C/A337F had a *K*_cat_ */K*_M_ value of 4.76 mm ^−1^ min^−1^, representing ≈1.66 folds that of mCPR‐T5H and 36.62 folds that of WT (Table 2). The relative levels of Fe^II^ in lysates from mCPR‐T5H‐T331C/A337F was 2.29, which was ≈56.85% higher than that of mCPR‐T5H after T5H catalyzed the reaction for 15 min (Figure 4e). This indicated that the electron‐receiving rate and T5H catalytic activity were further enhanced in mCPR‐T5H‐T331C/A337F.

In conclusion, the modification of residues at the terminal of the substrate channel could shorten the distance between the exit position of the substrate through the substrate channel and heme‐Fe, which accelerated the displacement of the substrate to water molecules in heme‐Fe^III^‐OH_2_, thereby increasing the electron‐receiving rate and T5H catalytic activity.

### 2.4 Enlarging Electron Supply by Fine‐Tuning NADPH Synthesis

**Figure 5:**
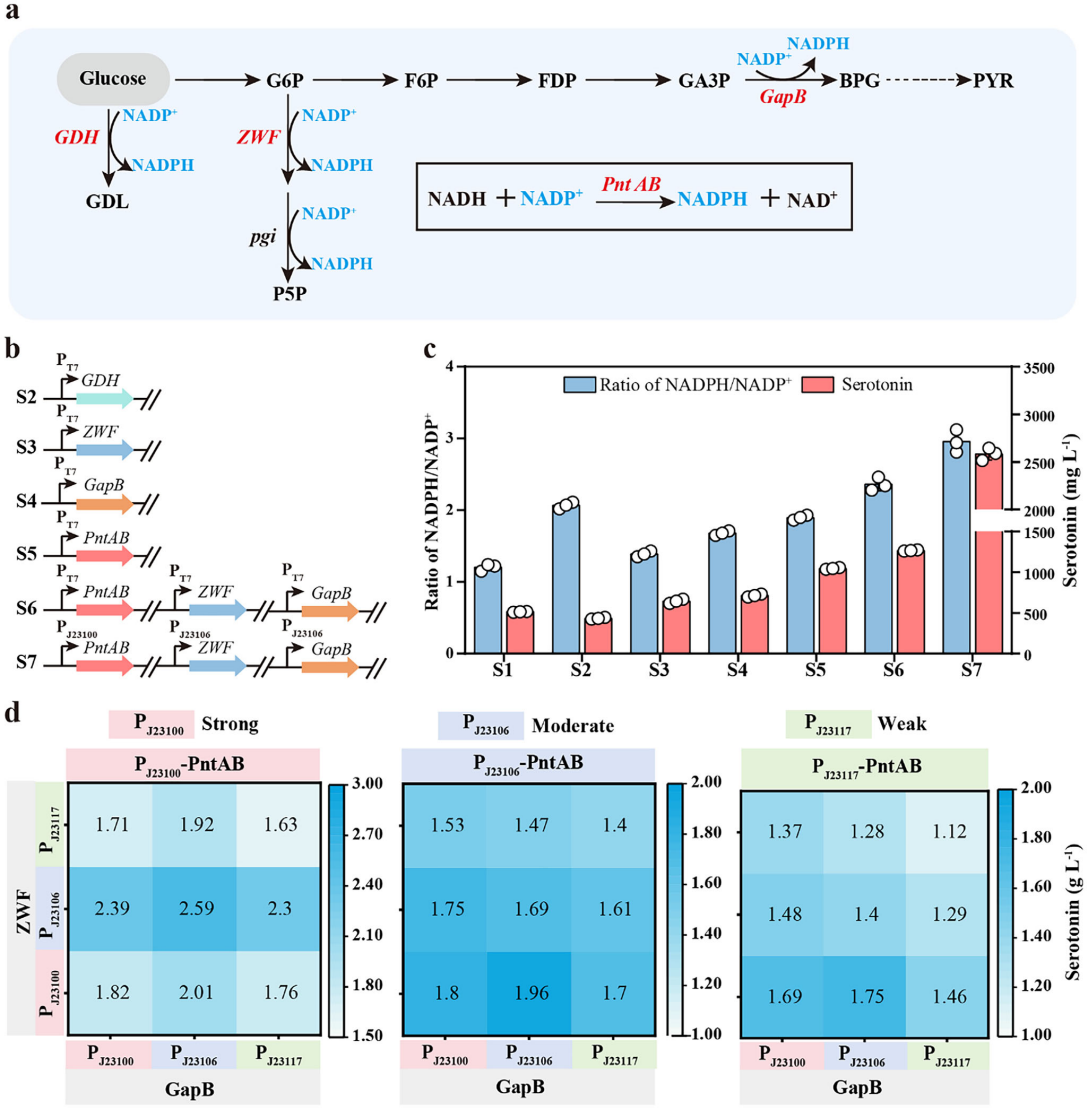
Fine‐tuning the expression of key enzymes for NADPH synthesis. a) Schematic of the engineering strategy to increase intracellular NADPH availability. b) Schematic of the gene overexpressing the key NADPH‐producing enzymes in the plasmid pACYCduet‐1. c) The ratio of NADPH/NADP^+^ and the serotonin production in recombinant strains. d) The serotonin production in recombinant strains with the expression of Pnt AB, GapB and ZWF under the control of constitutive promoters of different strengths (*P*_J23100,_ strong; *P*_J23106,_ moderate; *P*_J23117,_ weak). G6P, Glucose 6‐P; F6P, 6‐phosphate fructose; FDP, Fructose 1,6‐diphosphate; GA3P, Glyceraldehyde 3‐phosphate; BPG, Diphosphoglycerate; PYR, Pyruvic acid; GDL, Gluconolactone; P5P, Pentose 5‐P; *GDH*, gene encoding Glucose dehydrogenase; *ZWF* gene encoding glucose‐6‐phosphate 1‐dehydrogenase; *pgi*, gene encoding glucose‐6‐phosphate isomerase; *GapB*, gene encoding glyceraldehyde 3‐phosphate dehydrogenase; *PntAB*, gene encoding membrane‐bound transhydrogenase. The error bars indicate the standard deviation of three biological replicates (n = 3).Significance was analyzed using a t‐test. Statistical significance is indicated as * for *P* < 0.05 and ** for *P* < 0.01, respectively.

Although the catalytic efficiency (*K*_cat_ */K*_M_) of T5H in mCPR‐T5H‐T331C/A337F was 36.62‐fold higher than that in WT, the whole‐cell catalytic yield of serotonin in mCPR‐T5H‐T331C/A337F was only 2.89‐fold higher than that in WT. In the one‐step enzymatic reaction of T5H and CPR to generate serotonin, the stoichiometry shows that production of one molecule of serotonin requires the consumption of one molecule of the cofactor NADPH. In the pure enzyme‐catalyzed condition, at least 3 mm NADPH µm^−1^ CPR µm^−1^ T5H was required when the catalytic efficiency of CPR‐T5H was maximized; at least 5 mm NADPH µm^−1^ mCPR µm^−1^ T5H‐T331C/A337F was required when the catalytic efficiency of the mutant mCPR‐T5H‐T331C/A337F was maximized (Figure S7a, Supporting Information). The intracellular NADPH content and NADPH/NADP^+^ ratio were detected at different times during mCPR‐mT5H whole‐cell catalysis. At the initial stage (0 h) of the catalytic reaction, the NADPH content was about 5.63 µm L^−1^, and the ratio of NADPH/NADP+ ratio was ≈1.2, indicating that intracellular NADPH was relatively abundant at this time (Figure S7b, Supporting Information). At the final stage (12 h) of the catalytic reaction, the NADPH content was ≈2.65 µm L^−1^ and the ratio of NADPH/NADP^+^ was about 0.37, indicating that NADPH could not satisfy the requirement of intracellular metabolism. Exogenous addition of 2 mm NADPH significantly increased serotonin production in both WT and mCPR‐T5H‐T331C/A337F during T5H whole‐cell catalysis (Figure S7c, Supporting Information). Therefore, we hypothesized that the availability of the electron donor, NADPH, limited the efficient synthesis of serotonin in vivo. To increase the availability of NADPH, plasmids were constructed to overexpress key NADPH‐producing enzymes respectively, including glucose dehydrogenase (GDH), glucose‐6‐phosphate 1‐dehydrogenase (ZWF), glyceraldehyde 3‐phosphate dehydrogenase (GapB), and membrane‐bound transhydrogenase (PntAB) (**Figure** **5**a). These plasmids were subsequently introduced into S1 strain (mCPR‐T5H‐T331C/A337F) to construct strains S2, S3, S4 and S5, respectively (Figure 5b). As shown in Figure 5c, all four strains exhibited a significant increase in the intracellular NADPH/NADP^+^ ratio compared with the S1 strain. Moreover, the serotonin production of S3, S4, and S5 were 668.93, 732.82, and 1057.04 mg L^−1^, respectively, reflecting increases of ≈29.09%, 41.42%, and 103.99% compared to that of S1 (Figure 5c). However, the serotonin yield of S2 was 443.74 mg L^−1^, representing a significant decrease of ≈14.36% compared to that of S1. This reduction may be attributed to the accumulation of gluconolactone synthesized by GDH, which cannot be further catabolized, thereby negatively affecting cell growth and leading to diminished serotonin production. Then, a plasmid co‐expression PntAB, ZWF, and GapB under the control of the inducible T7 promoter was constructed (Figure 5b), and introduced into the S1 strain to construct strain S6, resulting in a serotonin production of 1274.03 mg L^−1^, representing an increase of ≈145.87% compared to that in S1 (Figure 5c). To further explore the synergistic effects of the three key enzymes involved in NADPH synthesis, we fine‐tuned the expression levels of PntAB, ZWF, and GapB using constitutive promoters of varying strengths. As shown in Figure 5b,d, the introduction of the co‐expression plasmid of *P*_J23100_ *‐PntAB‐P*_J23106_ *‐ZWF‐P*_J23106_ *‐GapB* into the S1 strain resulted in strain S7, which achieved the highest serotonin production at 2.59 g L^−1^ (molar yield 53.17%), representing an increase of ≈104% over S6. Furthermore, the intracellular NADPH/NADP^+^ ratio in S7 was significantly higher than that in S6 (Figure 5c). These results suggested that the enhancement of intracellular NADPH availability through the synergistic expression of the NADPH regeneration pathway can increase the production of serotonin synthesized by T5H.

### 2.5 Constructing a Cellular Factory for Efficient Serotonin Synthesis

**Figure 6:**
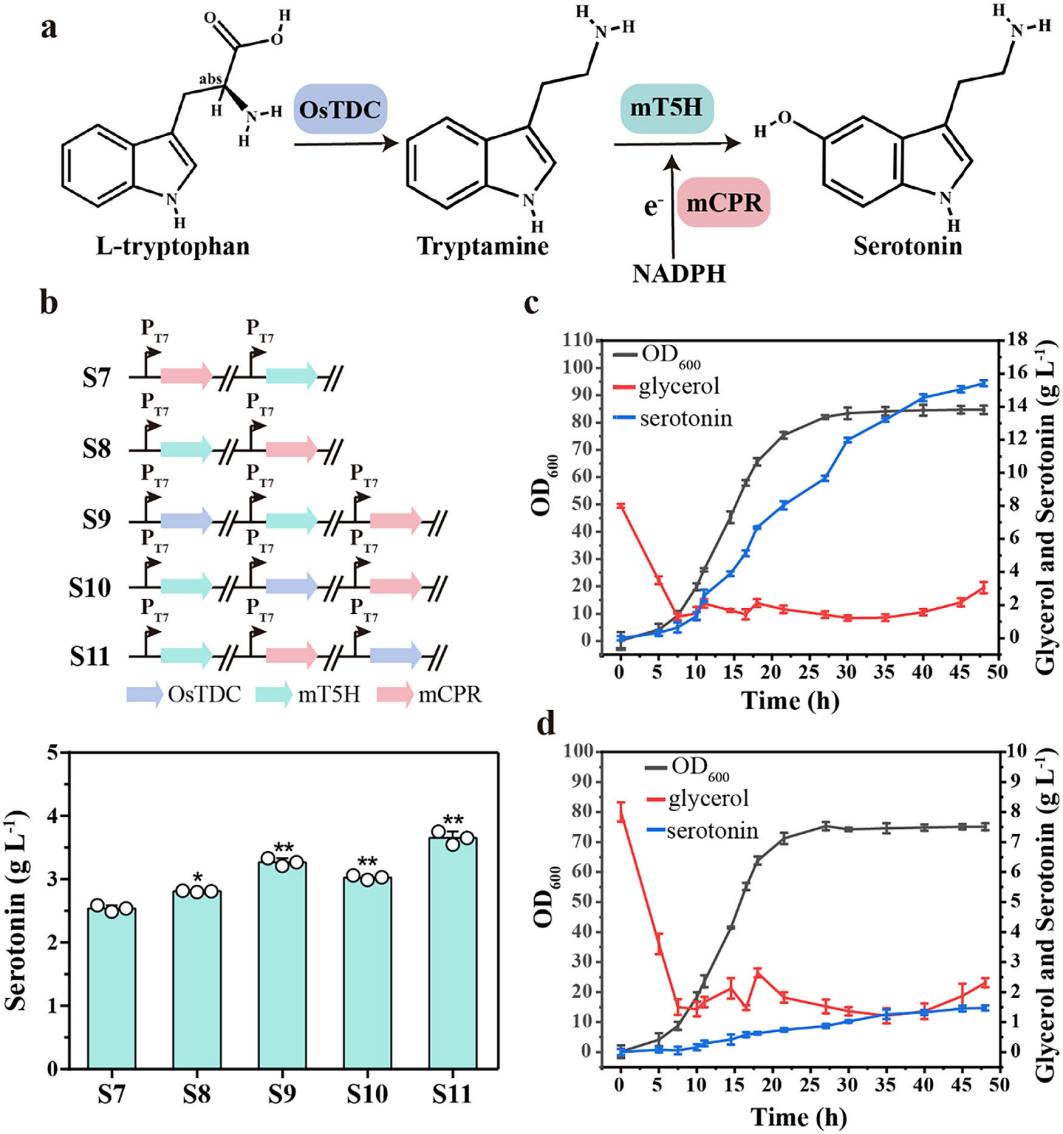
A cell factory constructed for high serotonin production. a) Serotonin biosynthetic pathway construct by OsTDC, mT5H (T5H‐331C337F) and mCPR (CPR‐683F690F) from L‐tryptophan. b) On the basis of S7, the production of serotonin under different expression systems of serotonin biosynthesis gene clusters. c) Fed‐batch fermentation of strain S11 in a 7.5 L fermenter. d) Fed‐batch fermentation of strain WT in a 7.5 L fermenter. The error bars indicate the standard deviation of three biological replicates (n = 3). Significance was analyzed using a t‐test. Statistical significance is indicated as * for *P* < 0.05 and ** for *P* < 0.01, respectively.

On the basis of increasing the whole‐cell catalytic yield of serotonin in S7, tryptophan decarboxylase (TDC) was employed to construct a cell factory for serotonin synthesis from L‐tryptophan. Three *TDC* genes^[^ ^9^, ^25^ ^]^ from *Catharanthus roseus* (*CrTDC*), *Galleria mellonella* (*GmTDC*), and *Oryza sativa Japonica Group* (*OsTDC*) with lower *K*_M_ values or higher enzyme activities were selected to construct strains capable of synthesizing tryptamine, respectively. OsTDC exhibited the highest serotonin yield among these TDCs, producing 123.13 mg L^−1^ (Figure S8a, Supporting Information), corresponding to a molar yield of 60.29% (g g^−1^ L‐tryptophan). Subsequently, based on S7, OsTDC, T5H‐T331C/A337F (mT5H), and CPR‐H683F/V690F (mCPR) were used to construct a serotonin biosynthesis strain from L‐tryptophan (**Figure** **6**a). Two strains, S7 and S8, were first constructed to evaluate the impact of various expression systems for *OsTDC*, *mT5H*, and *mCPR* on the pETduet‐1 plasmid. Serotonin production of S8 was 2.82 g L^−1^, which was ≈8.88% higher than that of S7 (Figure 6b). Then, the *P_T7_*‐*Os TDC* gene was then introduced into the anterior, middle, and posterior positions of *P_T7_‐mT5H* and *P_T7_‐mCPR*, respectively. The results showed that the S11 strain had a higher serotonin yield of 3.74 g L^−1^, which was ≈44.40% higher than that of S7 (Figure 6b). Although introducing OsTDC added an enzymatic reaction to the serotonin biosynthesis pathway, it also increased serotonin production. This increase may be attributed to a shift in the substrate for serotonin biosynthesis from tryptamine to L‐tryptophan, which alleviated the negative effects of tryptamine on cell growth (Figure S8b, Supporting Information).

The optimal strain, S11, was evaluated in a 7.5‐L bioreactor using fed‐batch fermentation for serotonin production. After 48 h of fermentation, the serotonin titer for S11 reached 15.42 g L^−1^, resulting in a production rate of 0.33 g L^−1^ h^−1^ and a yield of 0.77 g g^−1^ L‐tryptophan (Figure 6c). In contrast, the WT strain produced only 1.47 g L^−1^ of serotonin, with a production rate of 0.031 g L^−1^ h^−1^ and a yield of 0.074 g g^−1^ tryptamine (Figure 6d). Serotonin production by S11 was ≈10.49‐fold higher than that of the initial strain in the 7.5‐L reactor using fed‐batch fermentation. This also indicated that S11 was the most efficient serotonin‐producing strain, with the highest titer and production efficiency reported to date (Table S1, Supporting Information). Therefore, the serotonin‐producing strain developed in this study can be used for scaled‐up fermentation with a high yield. This lays the foundation for the industrial microbial production of serotonin.

## 3 Discussion

In recent years, P450s have attracted the attention of researchers because of their ability to introduce oxygen atoms into inactive C─H bonds during the biosynthesis of numerous natural products.^[^ ^8^, ^26^ ^]^ P450s must acquire electrons, either directly or indirectly from NAD(P)H, to achieve the reductive activation of inert oxygen and facilitate substrate mono‐oxidation.^[^ ^1^, ^20^ ^]^ Therefore, research on the ETF in P450 (and CPR) is important for improving P450 catalytic activity and analyzing its catalytic mechanism of P450. However, current tools and findings for studying ETF in P450 are relatively scarce. In this study, we reported that the systematic engineering of the ETF between T5H and CPR using an RNA biosensor, which effectively enhanced the catalytic activity of T5H and serotonin production. Given the unclear crystal structures and catalytic mechanisms of T5H and CPR, screening for highly active mutants using genetically encoded biosensors is a promising method. Herein, the construction of a serotonin RNA biosensor is more efficient using the modular aptameric biosensor method, because it starts with serotonin RNA aptamers and avoids the time‐consuming process and unpredictable results of screening biosensors de novo.^[^ ^27^ ^]^ Furthermore, combining a serotonin biosensor and fluorescence‐activated FACS can achieve the high‐throughput detection of intracellular serotonin. This study provides new insights into the development of novel biosensors for the evolution of strains and enzymes. Notably, serotonin RNA aptamers (5CG‐I and 5HR‐I) displayed three‐way junction (3WJ) conformations and excellent folding performance in vivo.^[^ ^14^ ^]^ Based on this, the serotonin biosensor could be further investigated for live‐cell imaging,^[^ ^16^ ^]^ which could contribute to detect the metabolic origins of serotonin across different cell types and to deal with serotonergic disorders.^[^ ^28^ ^]^ However, modular aptameric biosensors can only be used for detecting target metabolites and cannot perform regulatory functions. Serotonin RNA aptamers can be used to construct riboswitches that specifically recognize serotonin, thereby establishing a dynamic regulatory network for in vivo serotonin biosynthesis.^[^ ^27^, ^29^ ^]^

The steps involving electron transfer in the catalytic cycle of two‐component P450 (P450‐CPR) are i) the binding of NADPH at the FAD domain of CPR to acquire electrons, and electrons are subsequently transferred to the FMN domain; ii) the transfer of electrons from the FMN domain in CPR to the heme domain in P450 via ETP; and iii) the receiving of electrons in the heme.^[^ ^22b^ ^]^ In this study, we systematically improved the ETF from three perspectives based on electron transfer process. i) Enhancing electron transfer rate by engineering ETPs. The electron transfer rate is the transmission speed of the electron flux from the CPR (or reductase domain) to the heme domain in P450, which can be enhanced through rational design strategies for ETPs in one‐component P450s, such as alanine scanning and the introduction of aromatic amino acids.^[^ ^19a,b^ ^]^ However, the complexity of ETPs in two‐ or three‐component P450‐catalyzed systems may lead to suboptimal effects of these rational design strategies. Here, a putative ETP in the CPR of the two‐component P450 catalytic system was identified using eMAP, molecular docking and protein docking. The putative ETP was shortened by evolution and the intermediate site strategy, increasing the electron transfer rate in the hopping pathways, thereby significantly increasing the T5H catalytic activity. Moreover, the electronic behavior and electrochemical reactions should be further studied using technologies such as electron paramagnetic resonance and cyclic voltammetry.^[^ ^30^ ^]^ We have only considered the electron transfer process in the CPR. In contrast, the physical distance between T5H and CPR and the transfer of electrons from the terminal residue W660 of the putative ETP in CPR to the heme domain in T5H were not investigated. These key factors affecting electron transfer may contribute to our understanding of how to optimize ETF within two‐component P450 catalytic systems further. ii) Improving electron‐receiving rate by evolving the heme domain in T5H. The electron‐receiving rate is the speed of electron flux from the heme domain to heme‐Fe. In the catalytic cycle of P450s, heme serves as the final acceptor for electron transfer, and the residues surrounding heme significantly influence the catalytic activity of P450s.^[^ ^31^ ^]^ For example, C400H, a mutant of the heme axial ligand in P450 BM3, exhibited high activity in biocatalytic cyclopropanation.^[^ ^32^ ^]^ However, the influence of the heme domain of P450 on ETF remains unclear. Here, the heme domain of T5H was identified and evolved, resulting in a reduced distance between the end of the substrate channel and the heme‐Fe, which significantly increased the electron receiving rate and T5H catalytic activity. Although the process of electron receiving in P450 has been elucidated, the molecular mechanism of electron reception in the heme domain remains unknown and requires further exploration.^[^ ^33^ ^]^ For example, the energy profile during electron reception can be calculated using quantum mechanics/molecular mechanics (QM/MM).^[^ ^22b^ ^]^ iii) Enlarging electron supply by Fine‐tuning NADPH synthesis. The initial electron donor is NAD(P)H for the catalytic cycle of P450. Indeed, the supply of adequate NAD(P)H is one of the major limiting factors in the utilization of P450 enzymes in purified or extracted forms for industrial applications,^[^ ^8^ ^]^ which also limits the efficient synthesis of P450‐catalyzed natural products in microbial cell factories.^[^ ^6^ ^]^ Several strategies have been applied to enhance or substitute cofactors, including an alternative cofactor system constructed by the mediators cobalt(III) sepulchrate and zinc dust,^[^ ^34^ ^]^ light‐driven electron transfer,^[^ ^35^ ^]^ and altering cofactor preference.^[^ ^36^ ^]^ However, these strategies have poor applicability and lack economic viability. Introducing a cofactor regeneration system into the P450 catalytic cycle is a simple, fast and cost‐effective approach. Here, NADPH availability was increased by fine‐tuning the expression of key enzymes involved in NADPH synthesis, such as PntAB, GapB and ZWF. This significantly increased serotonin production and facilitated the construction of chassis cells with high NADPH production.

In addition to P450s, electron transfer is a major factor influencing the activity of metalloproteins, including cytochromes, blue copper proteins and ferredoxins.^[^ ^37^ ^]^ The long‐range transfer reactions of electrons in these metalloproteins are essential because they underpin oxidative phosphorylation, photosynthesis, and many intermediate metabolic reactions.^[^ ^38^ ^]^ Therefore, the ETF engineering proposed in this study contributes to accelerating intracellular energy conversion and many redox reactions.

## 4 Experimental Section

### Strains, Plasmids, and Medium

All constructed plasmids and strains are summarized in Tables S6 and S7 (Supporting Information), respectively. *E. coli* DH5α was used for plasmid construction and *E. coli* BL21 (DE3) was used for biosensor and protein expression. The serotonin biosensor screening plasmid used was pET‐30a, and the pETduet‐1 and pACYCduet‐1 plasmids were used for protein expression. Activation, expansion and IPTG induction of all strains were performed in LB culture. Biosensor screening was performed using M9 medium containing 5 mm MgSO4. Serotonin fermentation was performed using BCM‐1 (50 mm Tris‐HCl, pH 7.5, and 5% (v v^−1^) glycerol). If necessary, antibiotics were used at the following final concentrations: 100 µg mL^−1^ ampicillin, 10 µg mL^−1^ kanamycin and 50 µg mL^−1^ chloramphenicol.

### Construction and Screening of Serotonin Biosensor

The biosensor was constructed using 5CG‐I or 5HR‐I as serotonin aptamer, and different CM (Communication Module) sequences were added to construct a serotonin biosensor screening plasmid and the fluorescence detection method of the serotonin biosensor was performed as described by Porter et al.^[^ ^14^ ^]^ Serotonin biosensor‐related sequences were synthesized by Shanghai Sangon Biotech. The biosensor sequences were amplified using CM‐F and CM‐R and inserted into the pET‐30a plasmid by homologous recombination. The recombinant plasmids were introduced into *E. coli* BL21 (DE3). The recombinant strains were cultured in 50 mL conical flasks containing 10 mL LB media (kanamycin) at 37 °C and 200 rpm with shaking until OD_600_ = 0.6. Next, 0.5 mm IPTG was added and induced for 2 h at 37 °C. And, the cells were collected by centrifugation, washed three times with M9 medium (kanamycin), and resuspended in 300 µL M9 medium (kanamycin). The cell suspension was divided into two equal parts. One was added with 50 µm DFHBI‐1T in a final volume of 110 µL, and the other was added with 50 µm DFHBI‐1T and 1 mm ligand (L‐tryptophan, tryptophan, or serotonin) in a final volume of 110 µL. Notably, in the biosensor response to serotonin concentration experiments, one of the cell suspensions was added with 50 µm DFHBI‐1T and 0–5 mm serotonin to a final volume of 110 µL. Cells were incubated at 37 °C for 30 min, then 100 µL of each aliquot was pipetted into a 96‐well black microtiter plate and chilled on ice for 30 min. Finally, fluorescence detection was performed using a multifunctional enzyme plate at an excitation wavelength of 472 nm and an emission wavelength of 520 nm.

In vitro biosensor assay, the recombinant plasmid was first used as a template to generate the biosensor DNA sequence with the T7 promoter by a standard PCR reaction (primer: 5’ TAATACGACTCACTATAGCCCGGATAGCTCAGTCGG and 3’ TGGCGCCCGAACAGGGAC). Subsequently, the biosensor DNA was added directly to the in vitro transcription reaction and reacted at 37 °C for 2 h to synthesize RNA. The transcription reaction system (40 µL) consisted of 2 µL of T7 RNA polymerase (Takara), 4 µL of T7 RNA polymerase buffer, 16 µL of DNA, 4 µL of DTT (50 mm), 1 µL of RNAase inhibitor, 4 µL of NTP mixture, 8 µL enzyme‐free water. The RNA from the transcription reaction was divided directly into two aliquots, one of which was added with DFHBI‐1T (50 µm) and enzyme‐free water to a final volume of 110, and the other with DFHBI‐1T (50 µm) and enzyme‐free water and 0–3 mm serotonin. Finally, the fluorescence intensity in the serotonin in vitro reaction system was detected.

In the serotonin biosensor fluorescence assay, the fluorescence change value of the biosensor was calculated as follows:

(1) $$\mathrm{Relative}\;\mathrm{fluorescence}\;\mathrm{intensity}\;(\mathrm{EG})=\frac{\left[F\left(\mathrm{EG}\right)-F\left(\mathrm{NC}\right)\right]}{[F\left(\mathrm{blank}\right)-F\left(\mathrm{NC}\right)]}$$

(2) $$\mathrm{Relative}\;\mathrm{fluorescence}\;\mathrm{intensity}\;(\mathrm{black})=\frac{[F\left(\mathrm{blank}\right)-F\left(\mathrm{NC}\right)]}{[F\left(\mathrm{blank}\right)-F\left(\mathrm{NC}\right)]}$$

where F is green fluorescence intensity, EG is experimental group (the addition of 1 mm L‐tryptophan, tryptamine or serotonin), NC is negative control (an empty pET30a plasmid), blank is a blank control (the addition ddH_2_O).

In order to display the different of fluorescence intensity between 5CG‐CM3 and 5CG‐CM4 in the absence of serotonin, the fluorescence change value of the biosensor was calculated as follows:

(3) $$\mathrm{Fold}\;\mathrm{increase}\;(\mathrm{EG}\;\mathrm{or}\;\mathrm{black})=\frac{F\left(\mathrm{EG}\;\mathrm{or}\;\mathrm{black}\right)}{F\left(\mathrm{NC}\right)}$$

Dose‐response curves of biosensors were fitted by Boltzmann equation in Origin 2021.

### Random Mutation

After codon optimization of the amino acid sequences of T5H (GenBank accession no. AK071599, the transmembrane structural domain consisting of the first 37 residues in T5H was removed and a GST tag was added to the N‐terminal of T5H in this study) and CPR (AK099083), the corresponding DNA sequences were synthesized at Shanghai Sangon Biotech Co. Random mutant sequences of T5H or CPR were generated by error‐prone PCR. Error‐prone PCR kits were purchased from Beijing Solarbio Science & Technology (https://www.solarbio.com). The error‐prone PCR amplification primers for ‐T5H were RML‐T5H‐F1, RML‐T5H‐R1, RML‐T5H‐F2, RML‐T5H‐R2, RML‐T5H‐F3 and RML‐T5H‐R3 (Table S5, Supporting Information); and the error‐prone PCR amplification primers for CPR2 were RML‐CPR‐F1, RML‐CPR‐R1, RML‐CPR‐F2, RML‐CPR‐R2, RML‐CPR‐F3 and RML‐CPR‐R3. The mutation frequency was controlled by adding different volumes of Mut Enhance. Random mutant sequences were inserted into the pETduet‐1 plasmid (containing the P_T7_‐5CGCM4 sequence) by homologous recombination, and the recombinant plasmid was introduced into *E. coli* BL21 (DE3) by chemical transformation.

### High‐Throughput Screening

The recombinant strains were cultured in 50 mL conical flasks containing 10 mL LB media (ampicillin) at 37 °C and 200 rpm with shaking until OD_600_ = 0.6. Subsequently, 0.5 mm IPTG was added to induce the culture at 28 °C, 200 rpm for 18 h. Following this, 2 mm tryptamine was added and the catalytic reaction of the enzyme was carried out at 37 °C. Then, the organisms were collected by centrifugation, washed three times with M9 medium (ampicillin), and resuspended in 300 µL of M9 medium (ampicillin). Next, 50 μμ DFHBI‐1T was added, and the cell suspensions were incubated at 37 °C for 30 min and chilled on ice for 30 min. Finally, the cell suspensions were diluted to OD_600_ = 0.1‐0.3 in 5 mL flow tubes before fluorescence intensity was detected and categorized by flow cytometry.

### Fermentation Conditions

Whole‐cell catalysis in conical flasks and high‐performance liquid chromatography (HPLC) detection of serotonin were performed as described by Shen et al.^[^ ^39^ ^]^ In brief, the strains were cultured in 250 mL conical flasks containing 50 mL LB media (ampicillin) at 37 °C and 200 rpm with shaking until OD_600_ = 0.6. Subsequently, 0.5 mm IPTG was added to induce the culture at 28 °C, 200 rpm for 18 h. The strain was collected by centrifugation and resuspended in 50 mL conical flasks containing 5 mL BCM‐1 medium (50 mm Tris‐HCl, pH 7.5, 5% (v v^−1^) glycerol). Next, 15 mm tryptamine was added at an OD_600_ of 30 and incubated for 3 days at 37 °C and 200 rpm with shaking. Finally, serotonin productivity was measured using HPLC. During whole‐cell catalysis of the co‐expressed strains of key enzyme involved in NADPH synthesis and serotonin synthesis, 2% (v v^−1^) glucose and 25 mm tryptamine was added to BCM‐1. When TDC was introduced to construct the serotonin biosynthesis pathway, the substrate was changed to 25 mm L‐tryptophan.

For fed‐batch fermentation in a 7.5 L bioreactor, 2 L of fermentation medium was inoculated at an inoculum size of 10%. The fermentation medium contained 4 g L^−1^ (NH_4_)_2_HPO_4_, 13.5 g L^−1^ KH_2_PO_4_, 1.38 g L^−1^ MgSO_4_·7H_2_O, 1.7 g L^−1^ citric acid monohydrate, 1 g L^−1^ peptone, 2 g L^−1^ yeast extract, 8 g L^−1^ glycerol, 1% (v v^−1^) trace metal solution. The trace metal solution contained 0.1 g L^−1^ (NH_4_)_6_Mo_7_O_24_, 2.0 g L^−1^ CaCl_2_, 0.5 g L^−1^ MnSO_4_·4H_2_O, 10.0 g L^−1^ FeSO_4_·7H_2_O, 3.0 g L^−1^ CuSO_4_·5H_2_O, 5.3 g L^−1^ ZnSO_4_·7H_2_O, 0.2 g L^−1^ Na_2_B_4_O_7_·10H_2_O. In a 7.5‐L fermenter, the component of the feeding solution contained 600 g L^−1^ glycerol, 15 g L^−1^ MgSO_4_·7H_2_O, 2 g L^−1^ peptone, 4 g L^−1^ yeast extract. Fermentation was conducted at 37 °C, 20–30% dissolved oxygen (automatically adjusted with agitation rates) and pH 7.0 (adjusted with NH_3_·H_2_O). When the OD_600_ reached 10, 0.2 mm IPTG was added. At the same time the fermentation temperature was reduced to 32 °C. L‐tryptophan or tryptamine at a final concentration of 20 g L^−1^ was added to the bioreactor at a rate of 0.5–1 g L^−1^ h^−1^. The feeding solution was used to supplement glycerol during fermentation. Glycerol was detected as described by Kuhn et al.^[^ ^40^ ^]^

### Measurement of Enzyme Activity and the Content of NADPH in Cell Lysates

To measure T5H activity in the lysates, after 18 h of IPTG induction, the strains were collected by centrifugation and resuspended in 5 mL of Tris‐HCl (50 mm, pH 7.4). The cells were then lysed by an ultrasonic cell crusher. The cell suspension was kept in a mixture of ice and water, and the extraction cycle consisted of 3 s of disruption and 3 s of rest for a total of 100 cycles of 10 min. The lysed cell suspension was centrifuged at 10 000 rpm for 20 min at 4 °C. The total protein concentration in the supernatant was assayed using a modified Bradford Protein Assay Kit (Sangon Biotech Co., Ltd., Shanghai, China). The reaction mixture for the determination of enzymatic activity contained 2 mm NADPH, 0–3 mm tryptamine, 20% (v v^−1^) cell lysate (under conditions of normalization for total protein content), and Tris‐HCl was added to make the total volume of the reaction solution to 500 µL. The reaction mixture was subjected to 120 min at 37 °C and the serotonin content was detected by HPLC. The resulting data were fit to a Michaelis–Menten equation.

The reaction mixture for the determination of the content of NADPH contained 2 mm NADPH, 3 mm tryptamine, 20% (v v^−1^) cell lysate (under conditions of normalization for total protein content), and Tris‐HCl was added to make the total volume of the reaction solution to 500 µL. The reaction mixture was incubated at 37 °C for 30 min, 60 min, 90 min and 120 min, respectively, and then the NADPH content was measured according to the manufacturer's instructions (NADP^+^/NADPH Assay Kit with WST‐8, Beyotime Biotechnology, China).

### Purification of Enzymes

The genes of engineered His‐tagged T5H or CPR mutants (with deletion of 105 N‐terminal residues) were transformed into *E.coli* T7 express competent cells. The conditions for culture, induction and cell lysis of recombinant strains were as described above. The lysed cell suspension was centrifuged at 10 000 rpm for 20 min at 4 °C and followed by filtration with a 0.22 µm filter. The lysate was loaded onto a nickel affinity column (5 mL). Then, the hetero‐proteins were removed by washing buffer (50 or 100 mm imidazole, 300 mm NaCl and 50 mm NaH_2_PO_4_, pH 8.0) and the target proteins were rinsed and collected with 250 mm of imidazole (containing 300 mm NaCl and 50 mm NaH_2_PO_4_, pH 8.0). The collected target proteins were concentrated, desalted, and concentrated again to a final volume of 1mL with ultrafiltration tube. The purified proteins were analyzed by SDS‐PAGE.

### Measurement of Purified Enzyme Activity and Kinetic Parameters for the Selected CPR and T5H Mutants

The reaction mixture for the determination of enzymatic activity contained 1 µm purified CPR (or CPR mutants), 1 µm purified T5H (or T5H mutants), 5 mm NADPH, 3 mm tryptamine, and Tirs‐Hcl buffer (50 mm, pH 7.4) in a total volume of 500 µL. The reaction mixture for the determination of kinetic parameters contained 1 µm purified CPR (or CPR mutants), 1 µm purified T5H (or T5H mutants), 5 mm NADPH, 0–3 mm tryptamine, and Tirs‐Hcl buffer (50 mm, pH 7.4) in a total volume of 500 µL. The reaction mixture was subjected to 120 min at 37 °C and the serotonin content was detected by HPLC. The resulting data were fit to a Michaelis–Menten equation. The *K*_cat_ was calculated according to the equation *K*_cat_ = *V*_max_/C (C was the concentration of enzyme). And 1 U was defined as the amount of enzyme required to catalyze the production of serotonin from 1 µm tryptamine per minute.

### Molecular Docking and Molecular Dynamic Simulations

The 3D structure of NAPDH (Compound CID: 5884) and heme (Compound CID: 26945) was obtained from PubChem (https://pubchem.ncbi.nlm.nih.gov). The 3D structure of T5H and CPR was obtained from Uniport (https://www.uniprot.org/). The 3D structures of the mutant were generated by αFold2^[^ ^41^ ^]^ modeling. The GetBox plugin in PyMOL and AutoDock Tools were used for molecular docking.^[^ ^42^ ^]^ The calculation of molecular docking was performed as described by Zhang et al.^[^ ^43^ ^]^ In brief, the PDB files of the enzyme and substrate were imported in AutoDockTools‐1.5.6 and the corresponding pdbqt files were generated. Then the GetBox plug‐in was utilized to generate the 3D coordinates of the corresponding docking boxes and the coordinate data were incorporated into the parameter configuration file to execute the docking procedure. The molecular dynamics simulations were performed using Gromacs 2021.06 for 100 ns at 298.15 *K*. The AMBER99sb‐ildn force feld and the TIP3P water model were used. The root mean square deviation (RMSD) and root mean square fluctuation (RMSF) were calculated using the built‐in GROMACS program.

### Substrate Channel Analysis

Substrate channeling of T5H and its mutants was performed using the CAVER^[^ ^44^ ^]^ PyMOL plugin v3.0 (https://www.caver.cz/index.php?sid =121). The calculated results and superimposed comparisons of the substrate channels were obtained using PyMOL. The 3D coordinates of the center point on the end of the tunnel axis and the heme‐Fe were exported using PyMOL. The distance between the terminal point in the substrate channel and heme‐Fe was calculated by Equation (4).

(4) $$\mathrm{Distance}=\sqrt{{\left(X2-X1\right)}^{2}+{\left(Y2-Y1\right)}^{2}+{\left(Z2-Z1\right)}^{2}}$$

### Electron Transfer Pathway Analysis

The electron transport chains of CPR and its mutants were determined by eMAP^[^ ^23f^ ^]^ (https://emap.bu.edu). In brief, the Single Protein Analysis was selected in the eMAP website and the PDB file for the enzyme was imported. Then, tryptophan, tyrosine, phenylalanine, and histidine were selected for ETP calculation, and other parameters were left unchanged by default. Protein molecular docking was performed using GRAMM^[^ ^24^ ^]^ website (https://gramm.compbio.ku.edu/). In brief, the protein docking calculation was performed by importing the PDB files of CPR and T5H in the GRAMM website with CPR as the receptor and T5H as the ligand, and with the rest of the parameters remaining unchanged by default. The results of electron transport chain calculations and protein docking were analyzed using PyMOL.

### Calculation of Electron Transfer Efficiency

In enzymatic reactions, the electron transfer rate (*Κ*_ET_)^[^ ^19^, ^23^ ^]^ can be simplified to a distance‐dependent equation:

(5) $$k_{\mathrm{ET}}=A\left(0\right)e^{\left(-\beta R\right)}$$

where β is a measure of the ability to couple or superexchange electrons; R is the distance between the electron donor and the electron acceptor; and *Κ*_ET_ characterizes the rate of transferring electrons in units of S^−1^. According to Ivanov et al.^[^ ^45^ ^]^ this study was calculated with the values of A(0) = 2×10^12^ and β = 1.06 Å. R was calculated by adding the shortest distances between neighboring residues in the shortest pathway of electron transfer.

### Measurement of the Intracellular NADPH/NADP^+^ Ratio

The conditions for culture, induction and cell lysis of recombinant strains were as described above. And the assay for the ratio of NADPH/NADP^+^ according to the manufacturer's instructions (NADP^+^/NADPH Assay Kit with WST‐8, Beyotime Biotechnology, China).

### Detection of Relative Fe^II^ Level

The 1,10‐phenanthroline photometric method determined the relative level of Fe^II^.^[^ ^46^ ^]^ 20% acetic acid‐sodium acetate buffer (pH 4.2) was added to the cell lysates or the reaction solution (after 15 min of T5H enzyme catalysis) to precipitate proteins and extract ferrous ions. Subsequently, 40% ferrous ion extract, 0.08% 1,10‐phenanthroline (m/v) were added to 1 mL of the reaction system. After mixing the reaction solution, the reaction solution was allowed to stand for 15 min at 25 °C, and then the absorbance was detected at 510 nm by UV spectrophotometer. To characterize the variation of Fe^II^ levels before (0 min) and after (15 min) the enzyme‐catalyzed reaction, the relative level of Fe^II^ were calculated using the Fe^II^ content of mCPR‐T5H (or CPR‐T5H) under uncatalyzed reaction conditions as a control.

### Statistical Analysis

GraphPad Prism 6.0 was applied to analyzed experimental data. Dose‐response curves of biosensors and the Michaelis–Menten equation were fitted in Origin 2021. All experiments were independently conducted at least three times, and the results are expressed as means ± standard deviations (n = 3). Significance was analyzed using a t‐test. Statistical significance is indicated as * for *P* < 0.05 and ** for *P* < 0.01, respectively.

## Conflict of Interest

The authors declare no conflict of interest.

## Supporting information



Supporting Information

## Data Availability

The data that support the findings of this study are available from the corresponding author upon reasonable request.
