# Supplementary material for: Engineering Electron Transfer Flux between Cytochrome P450 Enzyme and P450 Reductase to Enhance Serotonin Production in Escherichia Coli
Source: Adv Sci (Weinh). 2025 May 23;12(30):e14859. doi: 10.1002/advs.202414859 (PMC12376664; doi:10.1002/advs.202414859)
Supplement: Supplementary file 1 — Supporting Information [file ADVS-12-e14859-s001.docx]

Supporting Information

Engineering electron transfer between cytochrome P450 enzyme and P450 reductase to enhance serotonin production in *Escherichia coli*

Wenzhao Xu, Pengling Wei, Lirong Chen, Ling Gao*, Xiaole Xia*


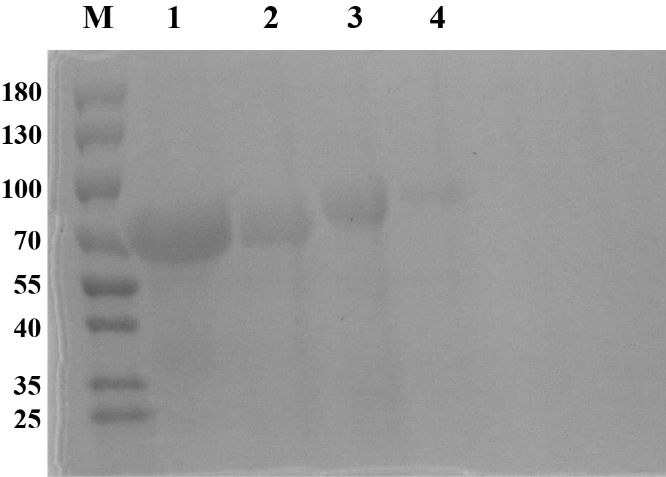


**Figure S1**. The SDS-PAGE analysis of the purified enzymes. The CPR protein was recovered after washing with wash buffer containing 50 mm imidazole (Lane 1) or 100 mm imidazole (Lane 2). The 105 residues at the N-terminus of the purified CPR were deleted. The T5H protein was recovered after washing with wash buffer containing 50 mm imidazole (Lane 3) or 100 mm imidazole (Lane 4). The 37 residues at the N-terminus of T5H were removed and its N-terminus was added with GST protein.


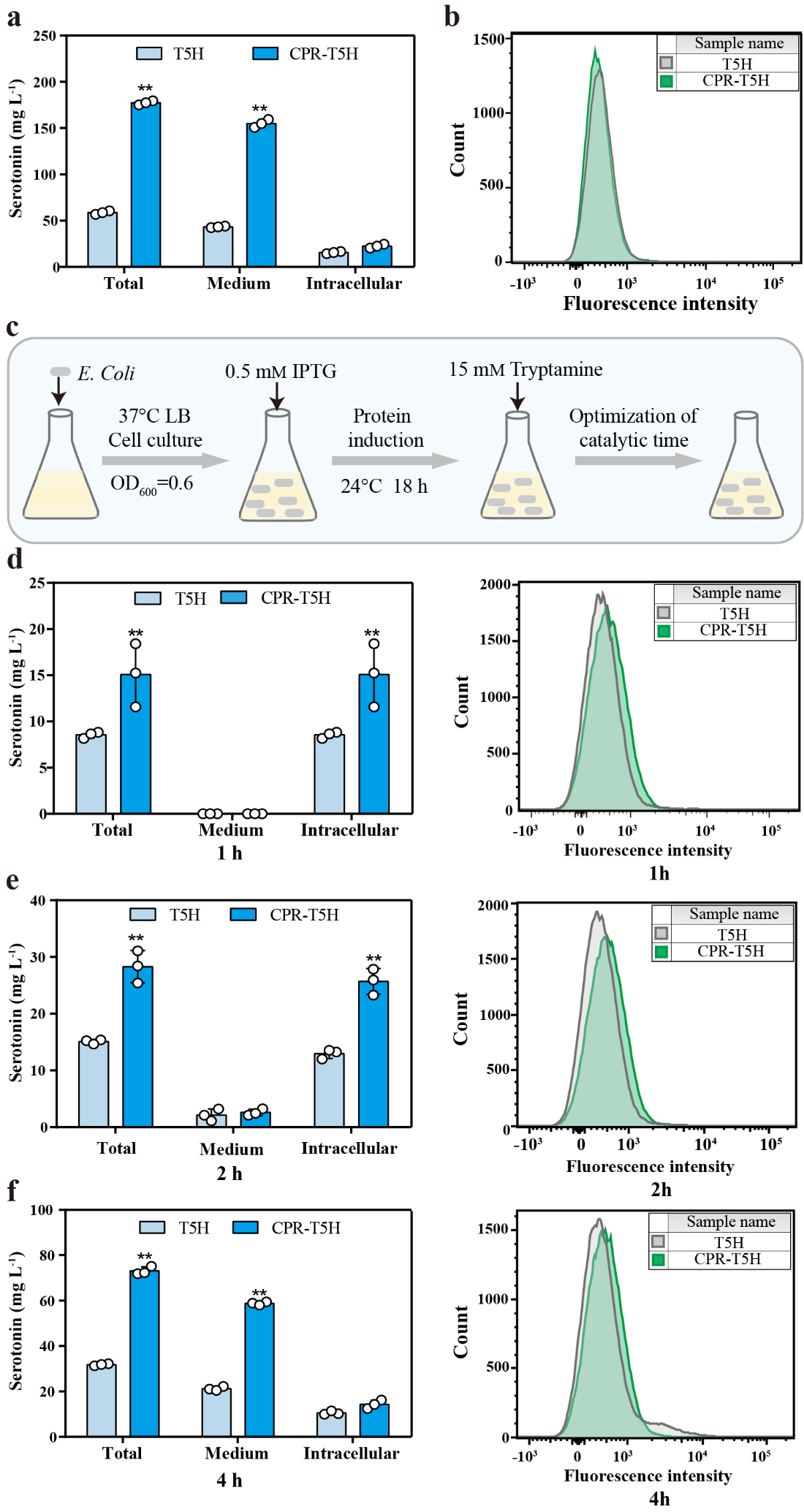


**Figure S2**. Optimization of high-throughput screening conditions for RNA biosensors. a) The serotonin yield in CPR-T5H and T5H. b) The fluorescence intensity at the single-cell level detected by flow cytometry. c) Schematic diagram of the optimized whole-cell catalytic process for high-throughput screening. d) The serotonin yield in CPR-T5H and T5H under whole-cell catalytic reaction for 1h and the fluorescence intensity at the single-cell level detected by FACS. e) The serotonin yield in CPR-T5H and T5H under whole-cell catalytic reaction for 2h and the fluorescence intensity at the single-cell level detected by FACS. f) The serotonin yield in CPR-T5H and T5H under whole-cell catalytic reaction for 4h and the fluorescence intensity at the single-cell level detected by FACS. The error bars indicate the standard deviation of three biological replicates (n = 3). Significance was analyzed using a t-test. Statistical significance is indicated as * for P<0.05 and ** for P<0.01, respectively.


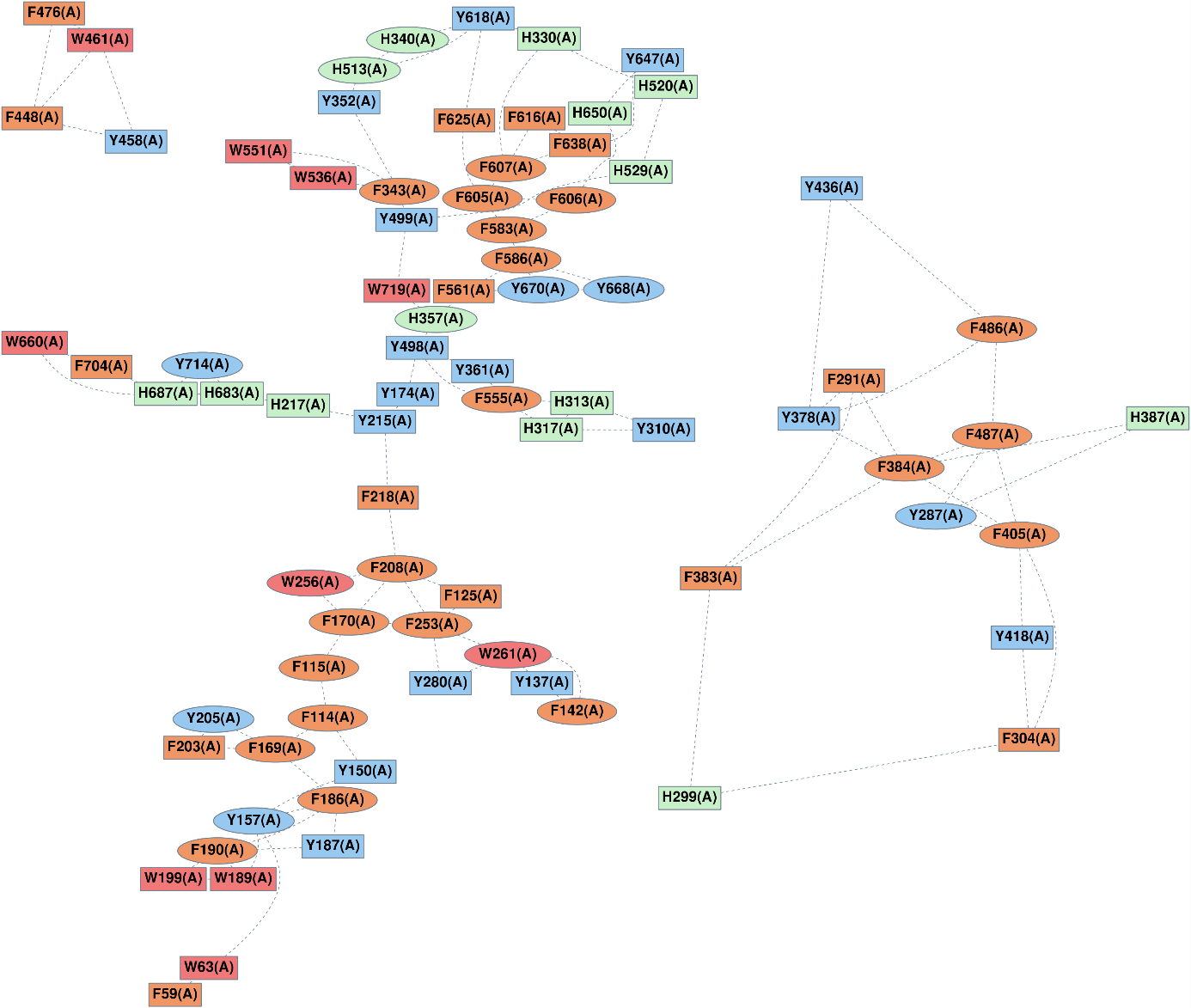


**Figure S3**. eMAP predicted all residues in CPR potentially involved in ETP


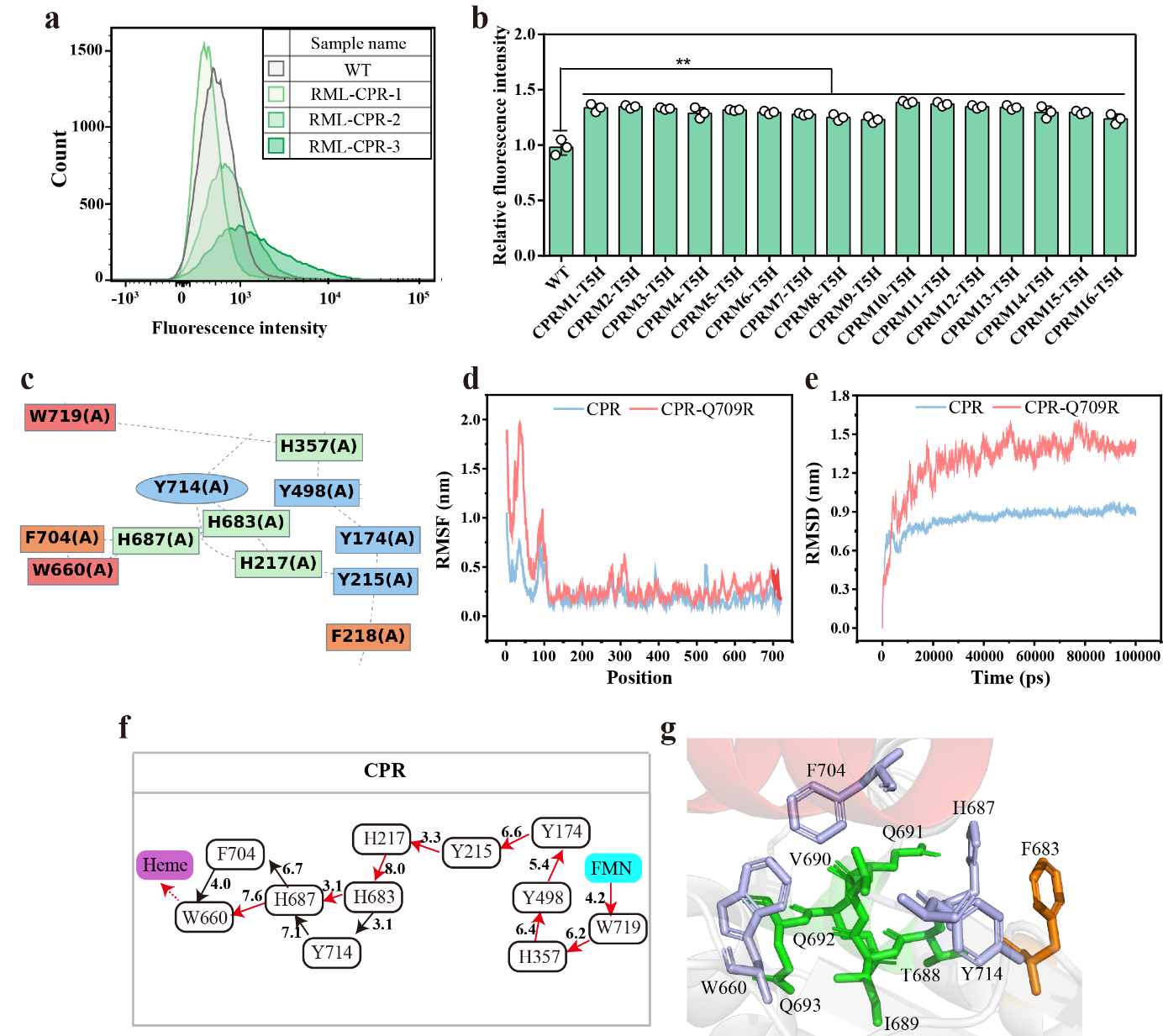


**Figure S4**. The high-throughput screening of random mutants in CPR and the engineering sites of the intermediate site strategy. a) Stepwise enrichment of high-fluorescence producers from the random mutation library of ETP in CPR (RML-CPR, numbers indicate screening rounds) using biosensor-based FACS-screening. b) Fluorescence intensity rescreening of CPR mutants by multifunctional enzyme marker. c) eMAP predicted the key residues in CPR-Q709R potentially involved in ETP (partial). d) RMSF analysis of CPR and CPR-Q709R. e) RMSD analysis of CPR and CPR-Q709R. f) The ETP of CPR was visualized in 2D. The shortest electron transfer directions (red arrows), the branching path of electron transfer directions (black arrows). g) The engineering sites of the intermediate site strategy between Y714 and W660. The error bars indicate the standard deviation of three biological replicates (n = 3). Significance was analyzed using a t-test. Statistical significance is indicated as * for P<0.05 and ** for P<0.01, respectively.


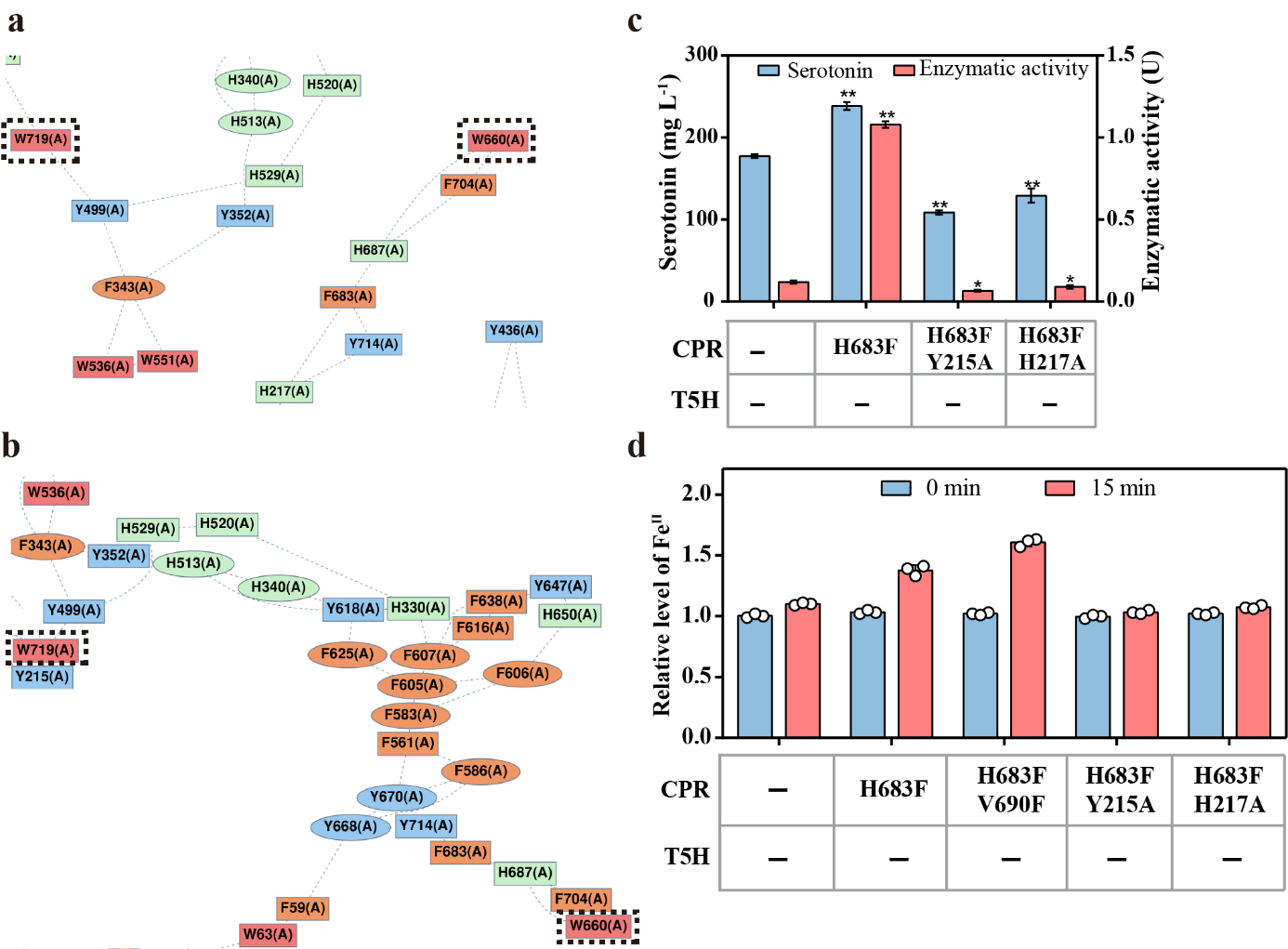


**Figure S5**. The effect of ETP disconnection or a substantial increase in the number of residues on serotonin production. a) eMAP predicted the key residues in CPR-H683F/Y215A potentially involved in ETP (partial). The residues in the black dashed box were the initial or terminal amino acids of the putative ETP in the CPR.b) eMAP predicted the key residues in CPR-H683F/H217A potentially involved in ETP (partial). c) The production of serotonin and enzyme activities of the CPR mutants. 1 U is defined as the amount of enzyme required to catalyze the production of serotonin from 1 μm tryptamine per minute. The reaction system of CPR-T5H (or their mutants) contained 1 μm purified CPR or CPR mutants (105 residues at the N-terminus of the purified CPR proteins were deleted), 1 μm purified T5H, 5 mm NADPH, 3 mm tryptamine, and Tirs-Hcl buffer (50 mm, pH 7.5) in a total volume of 500 μL. “-” indicated no mutant. d) The relative levels of Fe^II^ in cell lysates before and after 15 min of the catalytic reaction. The error bars indicate the standard deviation of three biological replicates (n = 3). Significance was analyzed using a t-test. Statistical significance is indicated as * for P<0.05 and ** for P<0.01, respectively.


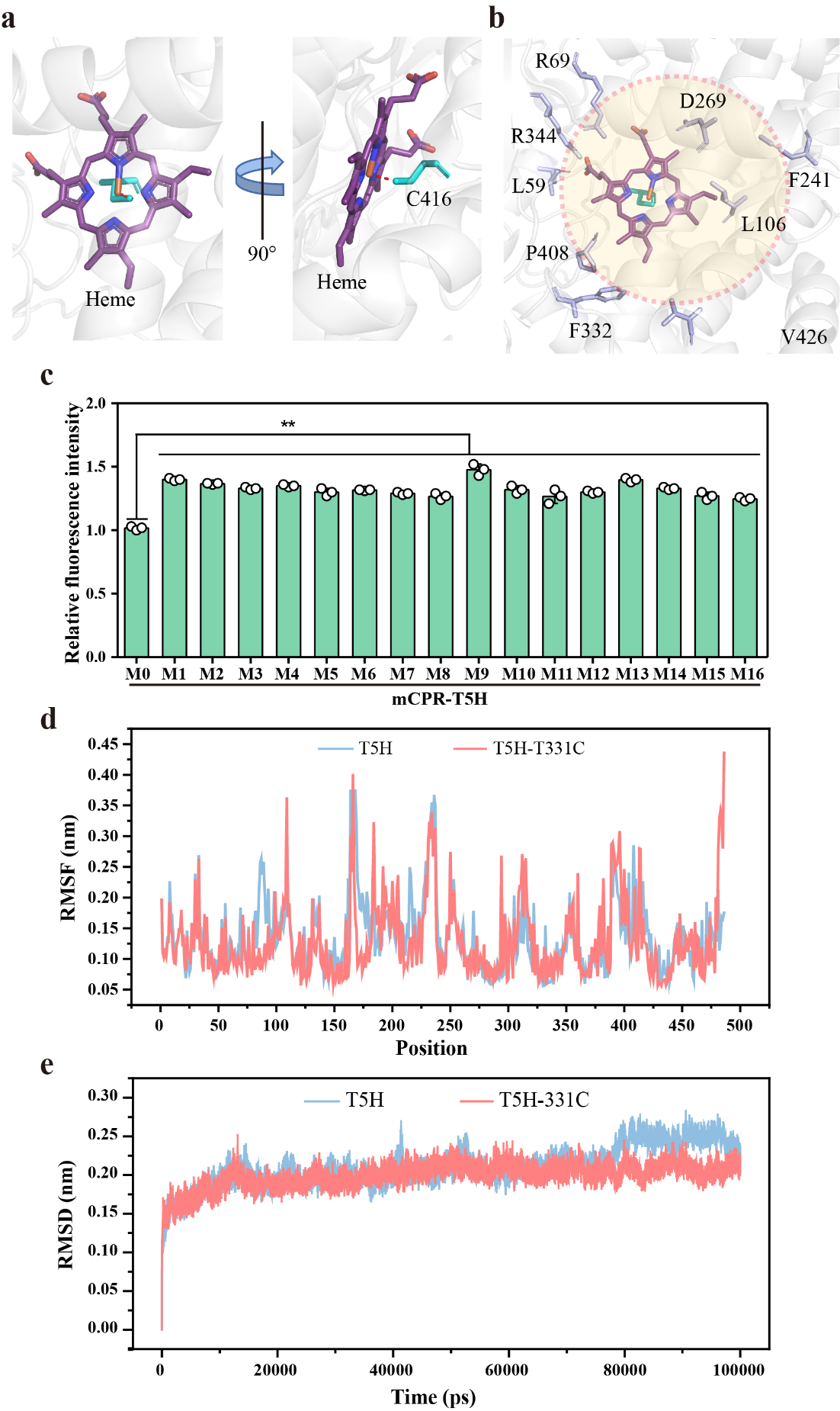


**Figure S6**. The evolution of the heme-binding region in T5H and molecular dynamics simulations of mutant. a) Display of the docking structure of heme and T5H via Autodock vina. The cysteine (C416, cyan) thiol group was ligated to the heme-iron (dark purple) to form the axial ligand. b) Residues of the heme-binding domain (5 Å around the heme binding site) in T5H (partial). c) Fluorescence intensity rescreening by multifunctional enzyme marker. M0 indicated no mutation (mCPR-T5H). d) RMSF analysis of T5H and T5H-T331C. e) RMSD analysis of T5H and T5H-T331C. The error bars indicate the standard deviation of three biological replicates (n = 3). Significance was analyzed using a t-test. Statistical significance is indicated as * for P<0.05 and ** for P<0.01, respectively.


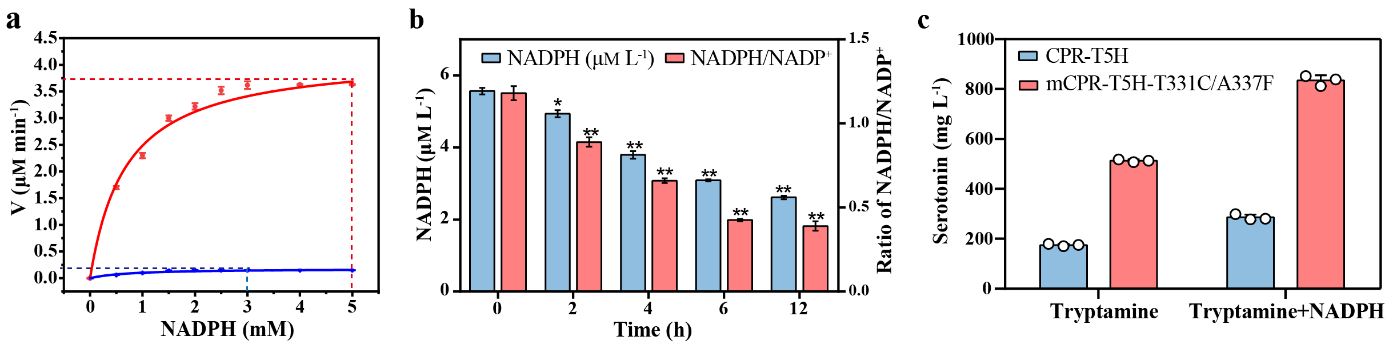


**Figure S7**. a) Enzyme kinetic curve for CPR-T5H (blue) and mCPR-mT5H (red). The 105 residues at the N-terminus of CPR and mCPR were removed for the extraction and purification of proteins. The reaction system contained 1 μm purified CPR (or CPR mutants), 1 μm purified T5H (or T5H mutants), 0-5 mm NADPH, 3 mm tryptamine, and Tirs-Hcl buffer (50 mm, pH 7.5) in a total volume of 500 μL. b) The content of NADPH and the ratio of intracellular NADPH/NADP^+^ at different times of mCPR-mT5H catalysis. c) The serotonin production of WT (CPR-T5H) and mCPR-T5H-T331C/A337F with 2 mm NADPH addition. The error bars indicate the standard deviation of three biological replicates (n = 3). Significance was analyzed using a t-test. Statistical significance is indicated as * for P<0.05 and ** for P<0.01, respectively.


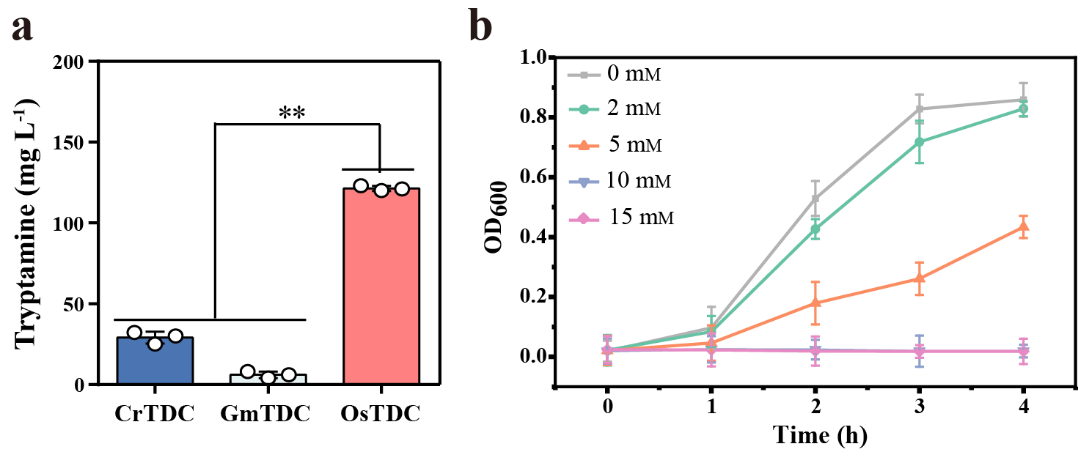


**Figure S8.** The construction of a cell factory for serotonin synthesis from L-tryptophan. b) Whole-cell catalysis of tryptamine production in tryptophan decarboxylases (TDCs), CrTDC, GmTDC and OsTDC were obtained from *Catharanthus roseus*, *Galleria mellonella* and *Oryza sativa Japonica Group*, respectively. c) The effect of 0-15 mm tryptamine on the growth of *E. coli* strain BL21. The error bars indicate the standard deviation of three biological replicates (n = 3). Significance was analyzed using a t-test. Statistical significance is indicated as * for P<0.05 and ** for P<0.01, respectively.

**Table S1** Overview of serotonin biosynthesis in microorganisms

| Strains | synthesis pathways | Titer (mg L^-1^) | Cultivation conditions | source or reference |
| --- | --- | --- | --- | --- |
| *E. Coli* | TDC | 35 | Addition of 5-hydroxytryptophan | Park et al., 2008^[1]^ |
| Yeast | TDC | 40 | Addition of galactose | Park et al., 2008^[1]^ |
| *E. Coli* | TDC and GST-Δ37T5H | 28 | Addition of L-tryptophan | Park et al., 2011^[2]^ |
| *E. Coli* BL21 (DE3) | GST-Δ37T5H and OsCPR2 | 250 | Addition of tryptamine | Park et al., 2013^[3]^ |
| *E. Coli* BL21 (DE3) | Stepwise synthesis: phenylalanine hydroxylase mutant with the tetrahydromonapterin regeneration pathway and TDC | 154.3 | Shake flask, fed-batch, addition of glucose | Mora-Villalobos et al., 2018^[4]^ |
| *E. Coli* NEBExpress | Purified enzymes: tryptophan  hydroxylase isoform 2 and aromatic amino acid decarboxylase | 87.6 | One pot, addition of tetrahydrobiopterin and L-tryptophan | Groaz et al., 2020^[5]^ |
| *E. Coli* BL21 (DE3) | Tryptophan hydroxylase with BH4 synthesis and regeneration pathway and dopa decarboxylase | 414.5±1.6 (molar yield  25.9%) | Shake flask, addition of L-tryptophan | Wang et al., 2022^[6]^ |
| *E. Coli*  MG1655 | Tryptophan hydroxylase with BH4 synthesis and regeneration pathway and TDC | 338 | Shake flask, addition of L-tryptophan | Shen et al., 2022^[7]^ |
|  |  | 1680 (molar yield  40.3%) | 5 L bioreactor, addition of L-tryptophan |  |
| *E. Coli* BL21 (DE3) | TDC, T5H and CPR mutants, and NADPH engineering | 3740 (molar yield 53.17%) | Shake flask, addition of L-tryptophan | This study |
|  |  | 15420 (molar yield 77%) | 7.5 L bioreactor, addition of L-tryptophan |  |

**Table S2** The performance of 5CG-CM4 biosensor in vivo and in vitro

|  | Fold increase | Basal output ^a)^ | linear range (mm Serotonin) | Sensitivity |
| --- | --- | --- | --- | --- |
| In vivo | 2.36-10.85 | 2.36±0.03 | 0.25-3.0 | 3.55 |
| In vitro | 1.43-7.94 | 1.43±0.01 | 0.5-2.0 | 5.32 |

^a)^ the fluorescence intensity at 0 mm serotonin addition (blank control)

**Table S3** The quantitative detection of serotonin concentration based on a serotonin biosensor

|  | Methods | Spiked (mm) | Detected (mm) | Recovery (%) ^a)^ |
| --- | --- | --- | --- | --- |
| In vivo | 5CG-CM4 | 0.75 | 0.87 | 116% |
|  | HPLC | 0.75 | 0.75 | 100% |
| In vitro | 5CG-CM4 | 0.75 | 0.79 | 105.33% |
|  | HPLC | 0.75 | 0.75 | 100% |

^a)^ Recovery (%) was equal to the detection value divided by the spiked value.

**Table S4** Substrate channel parameters of T5H and its mutants

|  | Length (Å) | Average radius (Å) | Radius at end of channel (Å) |
| --- | --- | --- | --- |
| T5H | 16.573 | 2.730 | 2.697 |
| T5H-V338L | 21.221 | 2.198 | 1.317 |
| T5H-A337F | 19.516 | 2.112 | 1.637 |
| T5H-A337T | 21.030 | 2.236 | 0.949 |
| T5H-V338A | 21.719 | 2.162 | 1.208 |

**Table S5** Distance between the terminal point in substrate channel and heme-Fe

|  | Three-dimensional coordinate (X, Y, Z) | Distance (Å) |
| --- | --- | --- |
| heme-Fe | (0.75, 2.64, 4.19) ^a)^ | - ^b)^ |
| T5H | (-0.30, 1.32, 0.32) ^c)^ | 4.22 |
| T5H-V338L | (1.66, 0.86, 2.72) ^c)^ | 2.48 |
| T5H-A337F | (1.42, 1.71, 1.86) ^c)^ | 2.59 |
| T5H-A337T | (-0.38, 1.33, 0.01) ^c)^ | 4.52 |
| T5H-V338A | (-1.71, 0.20, 0.83) ^c)^ | 4.82 |

^a)^ the three-dimensional coordinate of the iron ion (the center-of-mass) in the heme was calculated using PyMOL. ^b)^ “-” indicated not calculated. ^c)^ the three-dimensional coordinates of the terminal point (the center-of-mass) in substrate channel were calculated using PyMOL.

**Table S6** Plasmids used in the study.

| Plasmids | Description | Source |
| --- | --- | --- |
| pET-30a | T7 promoter, lac operator, and T7 terminator, Kan^r^ | Lab storage |
| pETduet-1 | T7 promoter, lac operator, and T7 terminator, Amp^r^ | Lab storage |
| pACYCduet-1 | T7 promoter, lac operator, and T7 terminator, Cm^r^ | Lab storage |
| pET-5CG-CM2 | pET-30a plasmid expressing the 5CG-CM2 biosensor from *E. coli* BL21 (DE3) | This study |
| pET-5CG-CM3 | pET-30a plasmid expressing the 5CG-CM3 biosensor from *E. coli* BL21 (DE3) | This study |
| pET-5CG-CM4 | pET-30a plasmid expressing the 5CG-CM4 biosensor from *E. coli* BL21 (DE3) | This study |
| pET-5CG-CM5 | pET-30a plasmid expressing the 5CG-CM5 biosensor from *E. coli* BL21 (DE3) | This study |
| pET-5HR-CM2 | pET-30a plasmid expressing the 5HR-CM2 biosensor from *E. coli* BL21 (DE3) | This study |
| pET-5HR-CM3 | pET-30a plasmid expressing the 5HR-CM3 biosensor from *E. coli* BL21 (DE3) | This study |
| pET-5HR-CM4 | pET-30a plasmid expressing the 5HR-CM4 biosensor from *E. coli* BL21 (DE3) | This study |
| pET-5HR-CM5 | pET-30a plasmid expressing the 5HR-CM5 biosensor from *E. coli* BL21 (DE3) | This study |
| pET-T5H | pET-30a plasmid expressing the *T5H* gene from *E. coli* BL21 (DE3) | This study |
| pET-CPR-T5H | pET-30a plasmid expressing the *CRP* and *T5H* gene from *E. coli* BL21 (DE3) | This study |
| pET-T5H-5CG-CM4 | pET-30a plasmid expressing the *T5H* gene and 5CG-CM4 biosensor from *E. coli* BL21 (DE3) | This study |
| pET-CPR-T5H-5CG-CM4 | pET-30a plasmid expressing the *CRP*, *T5H* gene and 5CG-CM4 biosensor from *E. coli* BL21 (DE3) | This study |
| pET-CPR-H683F/v690F-T5H (pET-mCPR-T5H) | pETduet-1 plasmid expressing the *CRP* (H683F/v690F) and *T5H* gene from *E. coli* BL21 (DE3) | This study |
| pET-CPR-H683F/H687A-T5H | pETduet-1 plasmid expressing the *CRP* (H683F/H687A) and *T5H* gene from *E. coli* BL21 (DE3) | This study |
| pET-CPR-H683F/Y215A-T5H | pETduet-1 plasmid expressing the *CRP* (H683F/Y215A) and *T5H* gene from *E. coli* BL21 (DE3) | This study |
| pET-CPR-H683F/H217A-T5H | pETduet-1 plasmid expressing the *CRP* (H683F/H217A) and *T5H* gene from *E. coli* BL21 (DE3) | This study |
| pET-mCPR-T5H-V338L | pETduet-1 plasmid expressing the *CRP* (H683F/v690F) and *T5H* (V338L) gene from *E. coli* BL21 (DE3) | This study |
| pET-mCPR-T5H-A337F | pETduet-1 plasmid expressing the *CRP* (H683F/v690F) and *T5H* (A337F) gene from *E. coli* BL21 (DE3) | This study |
| pET-mCPR-T5H-A337T | pETduet-1 plasmid expressing the *CRP* (H683F/v690F) and *T5H* (A337T) gene from *E. coli* BL21 (DE3) | This study |
| pET-mCPR-T5H-V338A | pETduet-1 plasmid expressing the *CRP* (H683F/v690F) and *T5H* (V338A) gene from *E. coli* BL21 (DE3) | This study |
| pET-mCPR-T5H-V338F | pETduet-1 plasmid expressing the *CRP* (H683F/v690F) and *T5H* (V338F) gene from *E. coli* BL21 (DE3) | This study |
| pET-mCPR-T5H-T331C | pETduet-1 plasmid expressing the *CRP* (H683F/v690F) and *T5H* (T331C) gene from *E. coli* BL21 (DE3) | This study |
| pET-mCPR-T5H-T331C/A337F  (pET-mCPR-mT5H) | pETduet-1 plasmid expressing the *CRP* (H683F/v690F) and *T5H* (T331C/A337F) gene from *E. coli* BL21 (DE3) | This study |
| pET-mCPR-T5H-A337F/V338L | pETduet-1 plasmid expressing the *CRP* (H683F/v690F) and *T5H* (A337F/V338L) gene from *E. coli* BL21 (DE3) | This study |
| pACYC-GDH | pACYCduet plasmid expressing the *gdh* gene from *E. coli* BL21 (DE3) | This study |
| pACYC-ZWF | pACYCduet plasmid expressing the *zwf* gene from *E. coli* BL21 (DE3) | This study |
| pACYC-GapB | pACYCduet plasmid expressing the *GapB* gene from *E. coli* BL21 (DE3) | This study |
| pACYC-PntAB | pACYCduet plasmid expressing the *PntAB* gene from *E. coli* BL21 (DE3) | This study |
| pACYC-PZG | pACYCduet plasmid expressing the *PntAB*, *zwf* and *GapB* gene from *E. coli* BL21 (DE3) | This study |
| pACYC-P_J23100_-PntAB-P_J23100_-ZWF-P_J23100_-GapB | pACYCduet plasmid expressing the *P_J23100_*-*PntAB*, *P_J23100_*-*zwf* and *P_J23100_*-*GapB* gene from *E. coli* BL21 (DE3) | This study |
| pACYC-P_J23100_-PntAB-P_J23100_-ZWF-P_J23106_-GapB | pACYCduet plasmid expressing the *P_J23100_*-*PntAB*, *P_J23100_*-*zwf* and *P_J23106_*-*GapB* gene from *E. coli* BL21 (DE3) | This study |
| pACYC-P_J23100_-PntAB-P_J23100_-ZWF-P_J23117_-GapB | pACYCduet plasmid expressing the *P_J23100_*-*PntAB*, *P_J23100_*-*zwf* and *P_J23117_*-*GapB* gene from *E. coli* BL21 (DE3) | This study |
| pACYC-P_J23100_-PntAB-P_J23106_-ZWF-P_J23100_-GapB | pACYCduet plasmid expressing the *P_J23100_*-*PntAB*, *P_J23106_*-*zwf* and *P_J23100_*-*GapB* gene from *E. coli* BL21 (DE3) | This study |
| pACYC-P_J23100_-PntAB-P_J23106_-ZWF-P_J23106_-GapB | pACYCduet plasmid expressing the *P_J23100_*-*PntAB*, *P_J23106_*-*zwf* and *P_J23106_*-*GapB* gene from *E. coli* BL21 (DE3) | This study |
| pACYC-P_J23100_-PntAB-P_J23106_-ZWF-P_J23117_-GapB | pACYCduet plasmid expressing the *P_J23100_*-*PntAB*, *P_J23106_*-*zwf* and *P_J23117_*-*GapB* gene from *E. coli* BL21 (DE3) | This study |
| pACYC-P_J23100_-PntAB-P_J23117_-ZWF-P_J23100_-GapB | pACYCduet plasmid expressing the *P_J23100_*-*PntAB*, *P_J23117_*-*zwf* and *P_J23100_*-*GapB* gene from *E. coli* BL21 (DE3) | This study |
| pACYC-P_J23100_-PntAB-P_J23117_-ZWF-P_J23106_-GapB | pACYCduet plasmid expressing the *P_J23100_*-*PntAB*, *P_J23117_*-*zwf* and *P_J23106_*-*GapB* gene from *E. coli* BL21 (DE3) | This study |
| pACYC-P_J23100_-PntAB-P_J23117_-ZWF-P_J23117_-GapB | pACYCduet plasmid expressing the *P_J23100_*-*PntAB*, *P_J23117_*-*zwf* and *P_J23117_*-*GapB* gene from *E. coli* BL21 (DE3) | This study |
| pACYC-P_J23106_-PntAB-P_J23100_-ZWF-P_J23100_-GapB | pACYCduet plasmid expressing the *P_J23106_*-*PntAB*, *P_J23100_*-*zwf* and *P_J23100_*-*GapB* gene from *E. coli* BL21 (DE3) | This study |
| pACYC-P_J23106_-PntAB-P_J23100_-ZWF-P_J23106_-GapB | pACYCduet plasmid expressing the *P_J23106_*-*PntAB*, *P_J23100_*-*zwf* and *P_J23106_*-*GapB* gene from *E. coli* BL21 (DE3) | This study |
| pACYC-P_J23106_-PntAB-P_J23100_-ZWF-P_J23117_-GapB | pACYCduet plasmid expressing the *P_J23106_*-*PntAB*, *P_J23100_*-*zwf* and *P_J23117_*-*GapB* gene from *E. coli* BL21 (DE3) | This study |
| pACYC-P_J23106_-PntAB-P_J23106_-ZWF-P_J23100_-GapB | pACYCduet plasmid expressing the *P_J23106_*-*PntAB*, *P_J23106_*-*zwf* and *P_J23100_*-*GapB* gene from *E. coli* BL21 (DE3) | This study |
| pACYC-P_J23106_-PntAB-P_J23106_-ZWF-P_J23106_-GapB | pACYCduet plasmid expressing the *P_J23106_*-*PntAB*, *P_J23106_*-*zwf* and *P_J23106_*-*GapB* gene from *E. coli* BL21 (DE3) | This study |
| pACYC-P_J23106_-PntAB-P_J23106_-ZWF-P_J23117_-GapB | pACYCduet plasmid expressing the *P_J23106_*-*PntAB*, *P_J23106_*-*zwf* and *P_J23117_*-*GapB* gene from *E. coli* BL21 (DE3) | This study |
| pACYC-P_J23106_-PntAB-P_J23117_-ZWF-P_J23100_-GapB | pACYCduet plasmid expressing the *P_J23106_*-*PntAB*, *P_J23117_*-*zwf* and *P_J23100_*-*GapB* gene from *E. coli* BL21 (DE3) | This study |
| pACYC-P_J23106_-PntAB-P_J23117_-ZWF-P_J23106_-GapB | pACYCduet plasmid expressing the *P_J23106_*-*PntAB*, *P_J23117_*-*zwf* and *P_J23106_*-*GapB* gene from *E. coli* BL21 (DE3) | This study |
| pACYC-P_J23106_-PntAB-P_J23117_-ZWF-P_J23117_-GapB | pACYCduet plasmid expressing the *P_J23106_*-*PntAB*, *P_J23117_*-*zwf* and *P_J23117_*-*GapB* gene from *E. coli* BL21 (DE3) | This study |
| pACYC-P_J23117_-PntAB-P_J23100_-ZWF-P_J23100_-GapB | pACYCduet plasmid expressing the *P_J23117_*-*PntAB*, *P_J23100_*-*zwf* and *P_J23100_*-*GapB* gene from *E. coli* BL21 (DE3) | This study |
| pACYC-P_J23117_-PntAB-P_J23100_-ZWF-P_J23106_-GapB | pACYCduet plasmid expressing the *P_J23117_*-*PntAB*, *P_J23100_*-*zwf* and *P_J23106_*-*GapB* gene from *E. coli* BL21 (DE3) | This study |
| pACYC-P_J23117_-PntAB-P_J23100_-ZWF-P_J23117_-GapB | pACYCduet plasmid expressing the *P_J23117_*-*PntAB*, *P_J23100_*-*zwf* and *P_J23117_*-*GapB* gene from *E. coli* BL21 (DE3) | This study |
| pACYC-P_J23117_-PntAB-P_J23106_-ZWF-P_J23100_-GapB | pACYCduet plasmid expressing the *P_J23117_*-*PntAB*, *P_J23106_*-*zwf* and *P_J23100_*-*GapB* gene from *E. coli* BL21 (DE3) | This study |
| pACYC-P_J23117_-PntAB-P_J23106_-ZWF-P_J23106_-GapB | pACYCduet plasmid expressing the *P_J23117_*-*PntAB*, *P_J23106_*-*zwf* and *P_J23106_*-*GapB* gene from *E. coli* BL21 (DE3) | This study |
| pACYC-P_J23117_-PntAB-P_J23106_-ZWF-P_J23117_-GapB | pACYCduet plasmid expressing the *P_J23117_*-*PntAB*, *P_J23106_*-*zwf* and *P_J23117_*-*GapB* gene from *E. coli* BL21 (DE3) | This study |
| pACYC-P_J23117_-PntAB-P_J23117_-ZWF-P_J23100_-GapB | pACYCduet plasmid expressing the *P_J23117_*-*PntAB*, *P_J23117_*-*zwf* and *P_J23100_*-*GapB* gene from *E. coli* BL21 (DE3) | This study |
| pACYC-P_J23117_-PntAB-P_J23117_-ZWF-P_J23106_-GapB | pACYCduet plasmid expressing the *P_J23117_*-*PntAB*, *P_J23117_*-*zwf* and *P_J23106_*-*GapB* gene from *E. coli* BL21 (DE3) | This study |
| pACYC-P_J23117_-PntAB-P_J23117_-ZWF-P_J23117_-GapB | pACYCduet plasmid expressing the *P_J23117_*-*PntAB*, *P_J23117_*-*zwf* and *P_J23117_*-*GapB* gene from *E. coli* BL21 (DE3) | This study |
| pET-mT5H-mCPR | pETduet-1 plasmid expressing the *T5H* (T331C/A337F) and *CRP* (H683F/v690F) gene from *E. coli* BL21 (DE3) | This study |
| pET-OsTDC | pETduet-1 plasmid expressing the *Oryza sativa Japonica Group TDC* gene from *E. coli* BL21 (DE3) | This study |
| pET-GmTDC | pETduet-1 plasmid expressing the *Galleria mellonella TDC* gene from *E. coli* BL21 (DE3) | This study |
| pET-CrTDC | pETduet-1 plasmid expressing the *Catharanthus roseus TDC* gene from *E. coli* BL21 (DE3) | This study |
| pET-osTDC-mT5H-mCPR | pETduet-1 plasmid expressing the *OsTDC*, *mT5H* and *mCPR* gene from *E. coli* BL21 (DE3) | This study |
| pET-mT5H-OsTDC-mCPR | pETduet-1 plasmid expressing the *mT5H, OsTDC* and *mCPR* gene from *E. coli* BL21 (DE3) | This study |
| pET-mT5H-mCPR-OsTDC | pETduet-1 plasmid expressing the *mT5H*, *mCPR* and *OsTDC* gene from *E. coli* BL21 (DE3) | This study |

**Table S7** Strains used in the study.

| Strains | Feature | Source |
| --- | --- | --- |
| *E. Coli* DH5α | Host for cloning plasmids | Lab storage |
| *E. Coli* BL21(DE3) | Host for expression plasmids | Lab storage |
| BL21-pET-5CG-CM2 | BL21(DE3) harboring plasmid pET-5CG-CM2, | This study |
| BL21-pET-5CG-CM3 | BL21(DE3) harboring plasmid pET-5CG-CM3 | This study |
| BL21-pET-5CG-CM4 | BL21(DE3) harboring plasmid pET-5CG-CM4 | This study |
| BL21-pET-5CG-CM5 | BL21(DE3) harboring plasmid pET-5CG-CM5 | This study |
| BL21-pET-5HR-CM2 | BL21(DE3) harboring plasmid pET-5HR-CM2 | This study |
| BL21-pET-5HR-CM3 | BL21(DE3) harboring plasmid pET-5HR-CM3 | This study |
| BL21-pET-5HR-CM4 | BL21(DE3) harboring plasmid pET-5HR-CM4 | This study |
| BL21-pET-5HR-CM5 | BL21(DE3) harboring plasmid pET-5HR-CM5 | This study |
| BL21-pET-T5H | BL21(DE3) harboring plasmid pET-T5H | This study |
| BL21-pET-CPR-T5H | BL21(DE3) harboring plasmid pET-CPR-T5H | This study |
| BL21-pET-T5H-5CG-CM4 | BL21(DE3) harboring plasmid pET-T5H-5CG-CM4 | This study |
| BL21-pET-CPR-T5H-5CG-CM4 | BL21(DE3) harboring plasmid pET-CPR-T5H-5CG-CM4 | This study |
| mCPR-T5H  (BL21-pET-CPR-H683F/v690F-T5H) | BL21(DE3) harboring plasmid pET-CPR-H683F/v690F-T5H | This study |
| BL21-pET-CPR-H683F/H687A-T5H | BL21(DE3) harboring plasmid pET-CPR-H683F/H687A-T5H | This study |
| BL21-pET-CPR-H683F/Y215A-T5H | BL21(DE3) harboring plasmid pET-CPR-H683F/Y215A-T5H | This study |
| BL21-pET-CPR-H683F/H217A-T5H | BL21(DE3) harboring plasmid pET-CPR-H683F/H217A-T5H | This study |
| BL21-pET-mCPR-T5H-V338L | BL21(DE3) harboring plasmid pET-mCPR-T5H-V338L | This study |
| BL21-pET-mCPR-T5H-A337F | BL21(DE3) harboring plasmid pET-mCPR-T5H-A337F | This study |
| BL21-pET-mCPR-T5H-A337T | BL21(DE3) harboring plasmid pET-mCPR-T5H-A337T | This study |
| BL21-pET-mCPR-T5H-V338A | BL21(DE3) harboring plasmid pET-mCPR-T5H-V338A | This study |
| BL21-pET-mCPR-T5H-V338F | BL21(DE3) harboring plasmid pET-mCPR-T5H-V338F | This study |
| BL21-pET-mCPR-T5H-T331C | BL21(DE3) harboring plasmid pET-mCPR-T5H-T331C | This study |
| S1  (BL21-pET-mCPR-mT5H) | BL21(DE3) harboring plasmid pET-mCPR-mT5H | This study |
| BL21-pET-mCPR-T5H-A337F/V338L | BL21(DE3) harboring plasmid pET-mCPR-T5H-A337F/V338L | This study |
| S2 | BL21(DE3) harboring plasmid pET-mCPR-mT5H and plasmid pACYC-GDH | This study |
| S3 | BL21(DE3) harboring plasmid pET-mCPR-mT5H and plasmid pACYC-ZWF | This study |
| S4 | BL21(DE3) harboring plasmid pET-mCPR-mT5H and plasmid pACYC-GapB | This study |
| S5 | BL21(DE3) harboring plasmid pET-mCPR-mT5H and plasmid pACYC-PntAB | This study |
| S6 | BL21(DE3) harboring plasmid pET-mCPR-mT5H and plasmid pACYC-PZG | This study |
| S6-2 | BL21(DE3) harboring plasmid pET-mCPR-mT5H and plasmid pACYC-P_J23100_-PntAB-P_J23100_-ZWF-P_J23100_-GapB | This study |
| S6-3 | BL21(DE3) harboring plasmid pET-mCPR-mT5H and plasmid pACYC-P_J23100_-PntAB-P_J23100_-ZWF-P_J23106_-GapB | This study |
| S6-4 | BL21(DE3) harboring plasmid pET-mCPR-mT5H and plasmid pACYC-P_J23100_-PntAB-P_J23100_-ZWF-P_J23117_-GapB | This study |
| S6-5 | BL21(DE3) harboring plasmid pET-mCPR-mT5H and plasmid pACYC-P_J23100_-PntAB-P_J23106_-ZWF-P_J23100_-GapB | This study |
| S7  (S6-6) | BL21(DE3) harboring plasmid pET-mCPR-mT5H and plasmid pACYC-P_J23100_-PntAB-P_J23106_-ZWF-P_J23106_-GapB | This study |
| S6-7 | BL21(DE3) harboring plasmid pET-mCPR-mT5H and plasmid pACYC-P_J23100_-PntAB-P_J23106_-ZWF-P_J23117_-GapB | This study |
| S6-8 | BL21(DE3) harboring plasmid pET-mCPR-mT5H and plasmid pACYC-P_J23100_-PntAB-P_J23117_-ZWF-P_J23100_-GapB | This study |
| S6-9 | BL21(DE3) harboring plasmid pET-mCPR-mT5H and plasmid pACYC-P_J23100_-PntAB-P_J23117_-ZWF-P_J23106_-GapB | This study |
| S6-10 | BL21(DE3) harboring plasmid pET-mCPR-mT5H and plasmid pACYC-P_J23100_-PntAB-P_J23117_-ZWF-P_J23117_-GapB | This study |
| S6-11 | BL21(DE3) harboring plasmid pET-mCPR-mT5H and plasmid pACYC-P_J23106_-PntAB-P_J23100_-ZWF-P_J23100_-GapB | This study |
| S6-12 | BL21(DE3) harboring plasmid pET-mCPR-mT5H and plasmid pACYC-P_J23106_-PntAB-P_J23100_-ZWF-P_J23106_-GapB | This study |
| S6-13 | BL21(DE3) harboring plasmid pET-mCPR-mT5H and plasmid pACYC-P_J23106_-PntAB-P_J23100_-ZWF-P_J23117_-GapB | This study |
| S6-14 | BL21(DE3) harboring plasmid pET-mCPR-mT5H and plasmid pACYC-P_J23106_-PntAB-P_J23106_-ZWF-P_J23100_-GapB | This study |
| S6-15 | BL21(DE3) harboring plasmid pET-mCPR-mT5H and plasmid pACYC-P_J23106_-PntAB-P_J23106_-ZWF-P_J23106_-GapB | This study |
| S6-16 | BL21(DE3) harboring plasmid pET-mCPR-mT5H and plasmid pACYC-P_J23106_-PntAB-P_J23106_-ZWF-P_J23117_-GapB | This study |
| S6-17 | BL21(DE3) harboring plasmid pET-mCPR-mT5H and plasmid pACYC-P_J23106_-PntAB-P_J23117_-ZWF-P_J23100_-GapB | This study |
| S6-18 | BL21(DE3) harboring plasmid pET-mCPR-mT5H and plasmid pACYC-P_J23106_-PntAB-P_J23117_-ZWF-P_J23106_-GapB | This study |
| S6-19 | BL21(DE3) harboring plasmid pET-mCPR-mT5H and plasmid pACYC-P_J23106_-PntAB-P_J23117_-ZWF-P_J23117_-GapB | This study |
| S6-20 | BL21(DE3) harboring plasmid pET-mCPR-mT5H and plasmid pACYC-P_J23117_-PntAB-P_J23100_-ZWF-P_J23100_-GapB | This study |
| S6-21 | BL21(DE3) harboring plasmid pET-mCPR-mT5H and plasmid pACYC-P_J23117_-PntAB-P_J23100_-ZWF-P_J23106_-GapB | This study |
| S6-22 | BL21(DE3) harboring plasmid pET-mCPR-mT5H and plasmid pACYC-P_J23117_-PntAB-P_J23100_-ZWF-P_J23117_-GapB | This study |
| S6-23 | BL21(DE3) harboring plasmid pET-mCPR-mT5H and plasmid pACYC-P_J23117_-PntAB-P_J23106_-ZWF-P_J23100_-GapB | This study |
| S6-24 | BL21(DE3) harboring plasmid pET-mCPR-mT5H and plasmid pACYC-P_J23117_-PntAB-P_J23106_-ZWF-P_J23106_-GapB | This study |
| S6-25 | BL21(DE3) harboring plasmid pET-mCPR-mT5H and plasmid pACYC-P_J23117_-PntAB-P_J23106_-ZWF-P_J23117_-GapB | This study |
| S6-26 | BL21(DE3) harboring plasmid pET-mCPR-mT5H and plasmid pACYC-P_J23117_-PntAB-P_J23117_-ZWF-P_J23100_-GapB | This study |
| S6-27 | BL21(DE3) harboring plasmid pET-mCPR-mT5H and plasmid pACYC-P_J23117_-PntAB-P_J23117_-ZWF-P_J23106_-GapB | This study |
| S6-28 | BL21(DE3) harboring plasmid pET-mCPR-mT5H and plasmid pACYC-P_J23117_-PntAB-P_J23117_-ZWF-P_J23117_-GapB | This study |
| BL21-pET-OsTDC | BL21(DE3) harboring plasmid pET-OsTDC | This study |
| BL21-pET-GmTDC | BL21(DE3) harboring plasmid pET-GmTDC | This study |
| BL21-pET-CrTDC | BL21(DE3) harboring plasmid pET-CrTDC | This study |
| S8 | BL21(DE3) harboring plasmid pET-mT5H-mCPR and plasmid pACYC-P_J23117_-PntAB-P_J23117_-ZWF-P_J23117_-GapB | This study |
| S9 | BL21(DE3) harboring plasmid pET-osTDC-mT5H-mCPR and plasmid pACYC-P_J23117_-PntAB-P_J23117_-ZWF-P_J23117_-GapB | This study |
| S10 | BL21(DE3) harboring plasmid pET-mT5H-OsTDC-mCPR and plasmid pACYC-P_J23117_-PntAB-P_J23117_-ZWF-P_J23117_-GapB | This study |
| S11 | BL21(DE3) harboring plasmid pET-mT5H-mCPR-OsTDC and plasmid pACYC-P_J23117_-PntAB-P_J23117_-ZWF-P_J23117_-GapB | This study |

**Table S8** The primers used for the constructed of plasmids.

| Primers | Sequence (5'-3') |
| --- | --- |
| CM-F | ggataacaattcccctctagaGCCCGGGATAGCTCAGTCGG |
| CM-R | gttatgctagttattgctcagcTGGCGCCCGAACAGGGAC |
| T5H-F | tataagaaggagatatacatATGTCCCCTATACTAGGTTATTGGAAA |
| T5H-R | TGGCAGCAGCCTAGGTTAATTAAACTTCAGACAGTTCTTCACCT |
| CPR-F | catcaccacagccaggatccGATGGAATCTAGCGCGGGC |
| CPR-R | cattatgcggccgcaagcttTTACCAAACATCACGCAGGTAGC |
| T5H-bioseneor-F | TTAACCTAGGCTGCTGCCATAATACGACTCACTATAGGGGAATT |
| T5H-bioseneor-R | GCCCCAAGGGGTTATGCTAG |
| RML-T5H-F1 | GATCTCCAAACCGGACCTG |
| RML-T5H-R1 | CCGCACCGTAGGTAGCAA |
| RML-T5H-F2 | GGGCGATCGTGATGAAGAT |
| RML-T5H-R2 | CACGGCGCTACGCTTTCA |
| RML-T5H-F3 | ACCCGGACTACAAACTGCTG |
| RML-T5H-R3 | GCTCGCCAGAGACACCTG |
| RML-CPR-F1 | GCCTGAAAAAAGAAAAAATCTCCCTGTTC |
| RML-CPR-R1 | TTCGTCCACAACTTTCGCAAC |
| RML-CPR-F2 | GGAATTTGATATTGCTGGTACTGGC |
| RML-CPR-R2 | AGCTGGAGATGGAGTAGTAGC |
| RML-CPR-F3 | GGTGATGCTAAAGGTATGGCG |
| RML-CPR-R3 | GCTTTTACCAAACATCACGCAG |
| CPR-V690F-F | CACCATCttcCAGGAACAGGGTAGCCTGGATT |
| CPR-V690F-R | GTTCCTGgaaGATGGTGTGCAGTACGCGGTGC |
| CPR-H687A-F | GTACTGgctACCATCGTTCAGGAACAGGGTAG |
| CPR-H687A-R | ACGATGGTagcCAGTACGCGGTGCACATCACG |
| CPR-Y215A-F | CGTCAGgctGAACACTTCAACAAAGTTGCGAAA |
| CPR-Y215A-R | AAGTGTTCagcCTGACGGTTACCCAGACCGAA |
| CPR-H217A-F | CCGTCAGTATGAAgctTTCAACAAAGTTGCGAAAGTTGTG |
| CPR-H217A-R | AagcTTCATACTGACGGTTACCCAGACCGAAG |
| T5H-V338L-F | GCGctgCCGCTGCTGGTGCCGCGTGAAAGCGT |
| T5H-V338L-R | ACCAGCAGCGGcagCGCCGGGTGCAGACGGAA |
| T5H-A337F-F | TCTGCACCCGttcGTGCCGCTGCTGGTGCCGC |
| T5H-A337F-R | GCACgaaCGGGTGCAGACGGAACGTTTCTTTG |
| T5H-A337T-F | TCTGCACCCGactGTGCCGCTGCTGGTGCCGC |
| T5H-A337T-R | GCACagtCGGGTGCAGACGGAACGTTTCTTTG |
| T5H-V338A-F | GCGgcaCCGCTGCTGGTGCCGCGTGAAAGCGT |
| T5H-V338A-R | ACCAGCAGCGGtgcCGCCGGGTGCAGACGGAA |
| T5H-V338F-F | GCGtttCCGCTGCTGGTGCCGCGTGAAAGCGT |
| T5H-V338F-R | ACCAGCAGCGGaaaCGCCGGGTGCAGACGGAA |
| T5H-T331C-F | CAAAGAAtgcTTCCGTCTGCACCCGGCGGTGC |
| T5H-T331C-R | GACGGAAgcaTTCTTTGATGATCGCACGCATG |
| GDH-F | catcaccacagccaggatccGATGTACCCGGATCTGAAAGGTA |
| GDH-R | ggcgcgccgagctcgaattcTTAGCCACGGCCCGCCTG |
| ZWF-F | catcaccacagccaggatccGATGGCGGTAACGCAAACA |
| ZWF-R | cattatgcggccgcaagcttTTACTCAAACTCATTCCAGGAACG |
| GapB-F | catcaccacagccaggatccGATGAAGGTAAAAGTAGCGATCAACG |
| GapB-R | cattatgcggccgcaagcttTTATACAGCAGACGGATGTTTCATT |
| PntAB-F | catcaccacagccaggatccGATGCGAATTGGCATACCAAG |
| PntAB-R | ggcgcgccgagctcgaattcTTAATTTTTGCGGAACATTTTCAGCATGC |
| PZG-F1 | agctctgtaaggtaccctcgTTATGCGACTCCTGCATTAGGA |
| PZG-R1 | cgagatcccgTTACTCAAACTCATTCCAGGAACG |
| PZG-F2 | gtttgagtaaCGGGATCTCGACGCTCTCC |
| PZG-R2 | gtg5gcagcagcctaggttaaTTATACAGCAGACGGATGTTTCATT |
| P_J23100_-PntAB-F | TTGACGGCTAGCTCAGTCCTAGGTACAGTGCTAGCATGCGAATTGGCATACCAAG |
| P_J23100_-PntAB-R | AGGACTGAGCTAGCCGTCAAAGAGCGTCGAGATCCCG |
| P_J23106_-PntAB-F | TttacggctagctcagtcctaggtatagtgctagcATGCGAATTGGCATACCAAG |
| P_J23106_-PntAB-R | aggactgagctagccgtaaAAGAGCGTCGAGATCCCG |
| P_J23117_-PntAB--F | TTGACAGCTAGCTCAGTCCTAGGGATTGTGCTAGCATGCGAATTGGCATACCAAG |
| P_J23117_-PntAB-R | AGGACTGAGCTAGCTGTCAAAGAGCGTCGAGATCCCG |
| P_J23100_-ZWF-F | TTGACGGCTAGCTCAGTCCTAGGTACAGTGCTAGCATGGCGGTAACGCAAACA |
| P_J23100_-ZWF-R | AGGACTGAGCTAGCCGTCAAAGTCGCATAACGAGGGTACC |
| P_J23106_-ZWF-F | TttacggctagctcagtcctaggtatagtgctagcATGGCGGTAACGCAAACA |
| P_J23106_-ZWF-R | aggactgagctagccgtaaAAGTCGCATAACGAGGGTACC |
| P_J23117_-ZWF--F | TTGACAGCTAGCTCAGTCCTAGGGATTGTGCTAGCATGGCGGTAACGCAAACA |
| P_J23117_-ZWF-R | TAGGACTGAGCTAGCTGTCAAAGTCGCATAACGAGGGTACC |
| P_J23100_-GapB-F | TTGACGGCTAGCTCAGTCCTAGGTACAGTGCTAGCATGAAGGTAAAAGTAGCGATCAACG |
| P_J23100_-GapB-R | AGGACTGAGCTAGCCGTCAATTACTCAAACTCATTCCAGGAACG |
| P_J23106_-GapB-F | TttacggctagctcagtcctaggtatagtgctagcATGAAGGTAAAAGTAGCGATCAACG |
| P_J23106_-GapB-R | aggactgagctagccgtaaATTACTCAAACTCATTCCAGGAACG |
| P_J23117_-GapB--F | TTGACAGCTAGCTCAGTCCTAGGGATTGTGCTAGCATGAAGGTAAAAGTAGCGATCAACG |
| P_J23117_-GapB-R | TAGGACTGAGCTAGCTGTCAATTACTCAAACTCATTCCAGGAACG |
| OsTDC-F | tataagaaggagatatacatATGGGTAGCCTGGATACCAACC |
| OsTDC-R | ttaccagactcgagggtaccTTAGTTCATCATTTCGGTGGTGG |
| GmTDC-F | tataagaaggagatatacatATGGAAGCGGGTGACTTCAA |
| GmTDC-R | ttaccagactcgagggtaccTTACGGTTTGTTGTTTTTGAAGGT |
| CrTDC-F | tataagaaggagatatacatATGGGTAGCATCGATTCTACCAA |
| CrTDC-R | ttaccagactcgagggtaccTTACGCTTCTTTCAGCAGATCATC |
| OsTDC-T5H-CPR-F | CGGCGTAGAGGATCGAGATACGGCCGCATAATCGAAAT |
| OsTDC-T5H-CPR-R | TTCGCGGGATCGAGATCGTTAGTTCATCATTTCGGTGGTGGT |
| T5H-OsTDC-CPR-F | GTAATCGTATTGTACACGGCC |
| T5H-OsTDC-CPR-R | GGTACCCTCGAGTCTGGT |
| T5H-CPR-OsTDC-F | CTCTGAAAGGAGGAACTATATCCGGTAATCGTATTGTACACGGCC |
| T5H-CPR-OsTDC-R | GCGTCCCATTCGCCAATCTTAGTTCATCATTTCGGTGGTGGT |

References

[1] M. Park, K. Kang, S. Park, K. Back, *Biosci Biotechnol Biochem.* **2008**, *72* (9), 2456.

[2] S. Park, K. Kang, S. W. Lee, M. J. Ahn, J. M. Bae, K. Back, *Appl Microbiol Biotechnol.* **2011**, *89* (5), 1387.

[3] S. Park, Y. S. Kim, S. G. Rupasinghe, M. A. Schuler, K. Back, *Bioprocess Biosyst Eng.* **2013**, *36* (3), 325.

[4] J. A. Mora-Villalobos, A. P. Zeng, *J Biol Eng.* **2018**, *12*, 3.

[5] G. S. Groaz A, Valer L,Rossetto D,Benedetti F,Guella G,Toparlak ÖD,Mansy SS, *Adv Biosyst.* **2022**, *4* (11), e2000118.

[6] Y. Wang, X. Chen, Q. Chen, N. Zhou, X. Wang, A. Zhang, K. Chen, P. Ouyang, *Microb Cell Fact.* **2022**, *21* (1), 47.

[7] P. Shen, S. Gu, D. Jin, Y. Su, H. Wu, Q. Li, J. Yang, W. He, J. Huang, F. Qi, *ACS Synth Biol.* **2022**, *11* (8), 2889.
